# Causal association between air pollution and allergic rhinitis, asthma: a Mendelian randomization study

**DOI:** 10.3389/fpubh.2024.1386341

**Published:** 2024-07-15

**Authors:** Juan Zhong, Weiye Li, Shasha Yang, Yifeng Shen, Xinrong Li

**Affiliations:** ^1^Chengdu University of Traditional Chinese Medicine, Chengdu, China; ^2^Chengdu Integrated TCM and Western Medicine Hospital/Chengdu First People’s Hospital, Chengdu, China; ^3^Otolaryngology Department, Guizhou University of Traditional Chinese Medicine, Guiyang, China; ^4^TCM Regulating Metabolic Diseases Key Laboratory of Sichuan Province, Hospital of Chengdu University of Traditional Chinese Medicine, Chengdu, China; ^5^Hospital of Chengdu University of Traditional Chinese Medicine, Chengdu, China

**Keywords:** air pollution, allergic rhinitis, asthma, Mendelian randomization, air pollutants

## Abstract

**Backgrounds:**

Observational studies suggest that air pollutants, including particulate matter and nitrogen compounds, could elevate asthma and allergic rhinitis health risks. Nevertheless, the exact nature of the causal relationship between air pollution and asthma and allergic rhinitis remains unknown. This study utilizes the Mendelian randomization (MR) technique to explore the potential causal links between air pollution components (PM_2.5_, PM_2.5–10_, PM_10_, NO₂, and nitrogen dioxide) and the incidence of allergic rhinitis and asthma.

**Methods:**

A MR study utilized summary statistics from GWAS that are publicly accessible. The inverse variance weighting (IVW) approach served as the foundational analysis technique. To ensure robustness, supplementary methodologies such as the weighted median, MR-Egger regression, simple mode, and weighted model were also applied. Heterogeneity was evaluated using Cochran’s Q test, and the presence of pleiotropy was determined through MR-Egger regression. The MR-PRESSO test was employed for outlier detection, and the analysis’s sensitivity was scrutinized via a leave-one-out strategy.

**Results:**

The IVW technique showed a strong correlation between PM10 and asthma (OR = 0.625, 95% CI = 0.396–0.988, *p* = 0.044). No significant associations were found between asthma and other air pollutants such as PM_2.5_, PM_2.5–10_, NO₂, or nitrogen dioxide. Similarly, allergic rhinitis showed no causal relationships with any studied air pollution metrics. Pleiotropy was absent in the findings. Sensitivity analyses, employing the leave-one-out method, confirmed the stability of these results, unaffected by individual single nucleotide polymorphisms (SNPs).

**Conclusion:**

This Mendelian randomization study establishes a causal link between PM10 exposure and asthma, suggesting that interventions to reduce air pollution may decelerate the adverse progression of asthma.

## 1 Introduction

Recognized as a significant reversible environmental determinant of premature mortality and morbidity, air pollution stands at the forefront of global health risks (1). This environmental hazard comprises a spectrum of particulates and gaseous pollutants, including but not limited to ozone, carbon monitrogen, sulfur dioxide, alongside nitrogenous compounds such as NO₂ and nitrogen dioxide (2). Among the array of pollutants, PM2.5 is distinguished as the most significant health threat. This is attributed to its finer composition, enabling deeper penetration into the lung tissue, and its association with a more harmful mix of chemicals (3). A growing body of epidemiological research underscores the association between exposure to air pollutants and a broad array of adverse health outcomes. These include, but are not limited to, respiratory and cardiovascular diseases (4, 5), cerebrovascular impairments (6), mental health issues (7), an increased risk of various cancers (8, 9) and respiratory diseases like allergic rhinitis (AR) and asthma (10). Allergic rhinitis and asthma are the most common allergic respiratory diseases. Since the respiratory tract can directly contact various air pollutants, both indoor and outdoor air pollutants are considered environmental risk factors for these diseases (11). Air pollutants, particularly ozone, particulate matter (PM), and diesel exhaust, have been demonstrated to increase the permeability of allergens in the respiratory mucosa and enhance the effects of allergen-induced airway inflammation (12). Furthermore, air pollutants make atopic patients more sensitive to antigens to which they are already sensitized (13). For example, Carlsten et al. observed that inhalation of diesel exhaust increases allergen-induced inflammation in the lower airways of atopic individuals (14).

Asthma and allergic rhinitis represent a considerable public health challenge and a worldwide health burden. AR is a widespread chronic disorder affecting the nasal lining, marked by symptoms and inflammatory and immunological dysfunctions. Manifestations include sneezing, itching in the nasal area, obstruction of airflow, and transparent nasal secretions, triggered by IgE-mediated responses (15). This condition has become a significant health issue globally (16), with an estimated 20% of the UK’s population affected (17). In China, a meta-analysis revealed that around 19% of adults and 22% of children are afflicted by AR (18), adversely influencing sleep quality, academic achievements, and life quality (19). Asthma, another chronic non-communicable respiratory illness affecting both minors and adults, arises from complex genetic and environmental interplays (20). It is identified by fluctuating respiratory symptoms and restricted airflow. Asthma’s prevalence has surged worldwide, now impacting about a third of the global populace and causing nearly 2.5 million deaths annually due to severe episodes (21). Collectively, asthma and AR affect a significant portion of the global population, over 20% (19), with projections indicating an upward trend. Hence, studying the relationship between air pollutants and allergic respiratory diseases holds significant clinical value, especially for devising crucial prevention and control strategies.

Research in the domain of environmental health has progressively uncovered the relationship between air pollution and respiratory conditions like asthma and allergic rhinitis. In a notable multicenter study designed as randomized, double-blind, and placebo-controlled, investigators assessed the effectiveness of air purifiers equipped with high-efficiency particulate air (HEPA) filters on adults diagnosed with AR. Findings from this investigation confirmed that utilizing air purifiers with HEPA filters led to a substantial decrease in the medication usage for individuals affected by AR triggered by HDM, alongside a marked reduction in the levels of indoor PM2.5 and allergic airway conditions relieving (22). In a parallel effort, a randomized, double-blind clinical trial conducted in China with 90 participants who had allergic rhinitis and were sensitive to Artemisia pollen demonstrated significant improvements upon using high-efficiency air purifiers (23). A separate cohort study brought to light the association between slight increases in exposure to traffic-related air pollutants, such as NO2 and PM2.5, during the year of birth and the subsequent development of asthma by the age of 7 (24). Further, an investigation by Schildcrout JS and his team (25), which analyzed asthma exacerbations among children in eight cities, found that increases in carbon monitrogen dioxideide and nitrogen dioxide levels were directly linked to exacerbations of asthma symptoms, with sulfur dioxide levels also showing a marginal association.

Despite these insights, observational studies are often challenged by methodological limitations, such as the risk of homologous or reverse causation, which may skew results due to unseen confounders or reverse causality. To surmount these challenges and solidify the causal inference, RCTs are advocated as the most reliable method to eliminate confounding variables typical of observational studies. Yet, the specific exploration of the nexus between air pollution, allergic rhinitis, and asthma through RCTs remains a significant gap in current research. Mendelian randomization (MR) (26) is increasingly recognized as an invaluable method for determining the veracity of observed correlations against causal hypotheses, adeptly navigating through the pitfalls of confounding variables and reverse causation that frequently complicate observational research. MR employs genetic variations, specifically SNPs, as instrumental variables, facilitating analyses that closely resemble the structure of RCTs without their logistical constraints. This resemblance is due to the random allocation of alleles during meiosis, analogous to the randomization process in RCTs, thereby simulating a natural experiment. MR capitalizes on the principle (27) that genetic variants are distributed randomly across populations, which theoretically eliminates any selection bias related to environmental exposures, such as air pollution. This framework ensures that the association between genetic markers and exposure to environmental factors is not confounded, providing a clearer path to establishing causal relationships. Furthermore, MR can significantly diminish the influence of potential confounding variables, including age, gender, and lifestyle choices, by isolating the direct relationship between genetic predispositions and health outcomes like allergic rhinitis and asthma. By doing so, MR offers a more solid foundation for causal inference than traditional observational studies. Thus, leveraging MR to investigate the link between air pollution and allergic diseases allows for overcoming the inherent limitations of observational studies, offering more reliable evidence of causality. This study utilizes a comprehensive collection of publicly available GWAS data, with allergic rhinitis and asthma as endpoints, focusing on exposure to PM2.5, PM2.5–10, PM10, NO₂, and nitrogen dioxide, to delve deeper into the potential causal connections between air pollution and these allergic conditions while minimizing the effects of confounding factors.

## 2 Materials and methods

### 2.1 Study design

**Figure 1:**
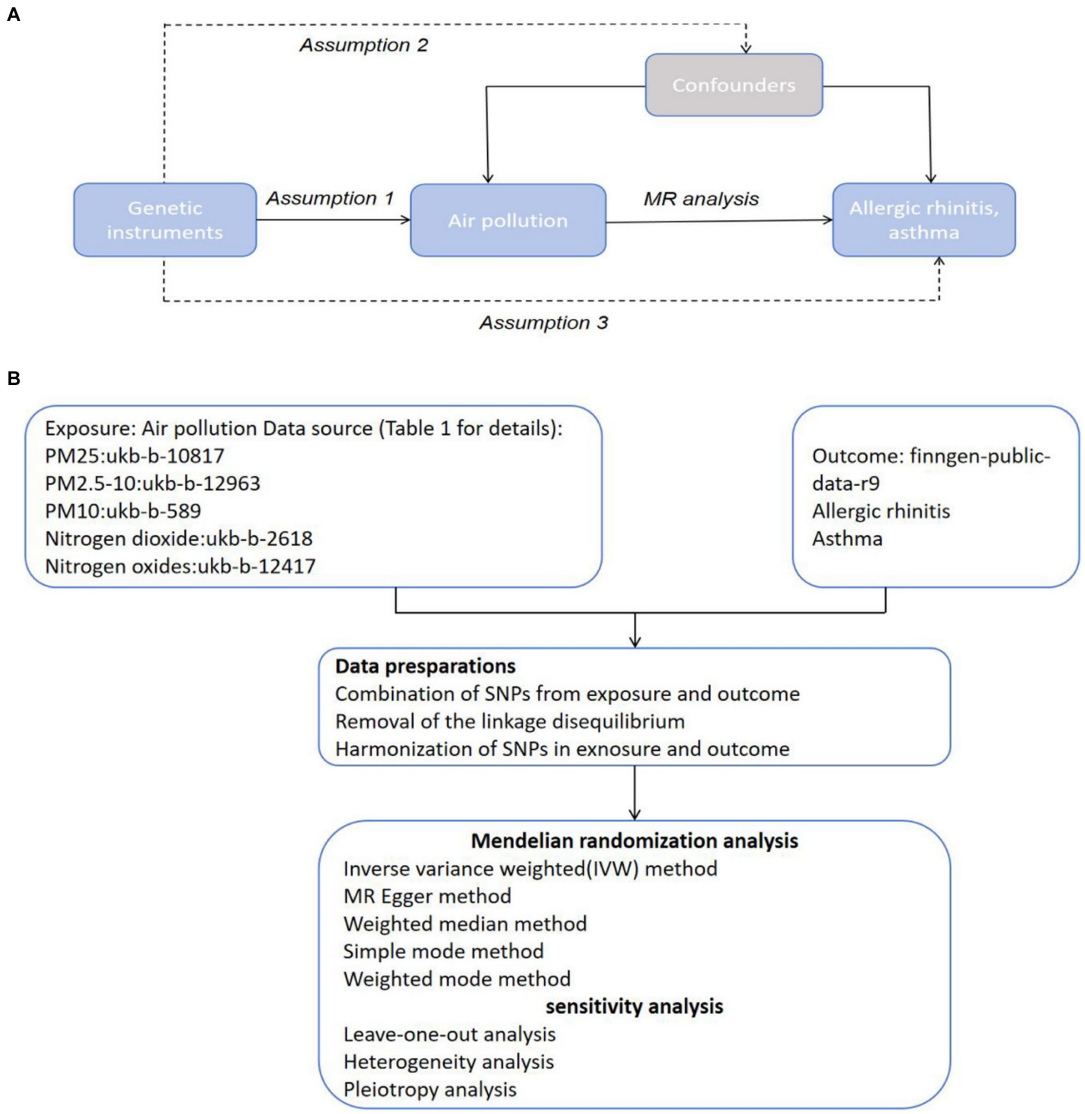
Hypothesis and Conceptual Framework Overview. **(A)** Mendelian Randomization Hypothesis Diagram: SNPs related to air pollution as genetic instruments for analyzing the causal influence of air pollution on frailty. Lines with arrows depict the association of SNPs with exposure, indicating mediation of the effect on outcome solely through exposure. Dashed lines show independence of SNPs from confounders affecting the results. **(B)** Methodological Blueprint for Mendelian Randomization Analysis.

Our investigation utilizes a MR framework, anchored on three fundamental principles: (1) Instrumental variables must be associated with the exposure; (2) They should not be linked to the outcomes through any confounding pathways; and (3) Their influence on the outcomes should be mediated exclusively through the exposure, not directly (as illustrated in Figure 1A). In our study, the focal exposure is air pollution, encompassing PM_2.5_, PM_2.5–10_, PM_10_, NO₂, and nitrogen dioxide, with the instrumental variables being SNPs that have a strong association with air pollution levels. The outcomes under investigation are allergic rhinitis and asthma. Employing a two-sample MR approach, we aim to elucidate the causal dynamics between air pollution and the incidence of allergic rhinitis and asthma. Figure 1 delineates the procedural blueprint of this Mendelian randomization study.

### 2.2 Data sources

Table 1 elaborately enumerates the sources of data utilized in this analysis. For the exposure variables, we selected various air pollutants, including PM_2.5_, PM_2.5–10_, PM_10_, nitrogen dioxide, and nitrogen oxides. The comprehensive data regarding these pollutants were sourced from the UK Biobank, an extensive cohort study comprising over half a million participants from the United Kingdom. This study has made available detailed information on phenotypes, genetic data, and genome-wide genotyping (28). The GWAS summary datasets specifically for PM2.5 (GWAS ID: ukb-b-10817), PM2.5–10 (GWAS ID: ukb-b-12963), PM10 (GWAS ID: ukb-b-589), nitrogen dioxide (GWAS ID: ukb-b-2618), and nitrogen oxides (GWAS ID: ukb-b-12417) included participant counts of 423,796, 423,796, 455,314, 456,380, and 456,380, respectively. It is noted that all participants had provided informed consent for their inclusion in the original studies from which this data was compiled.

**Table 1:** Overview of exposures data sources in this two-sample MR study.

Exposures		Dataset	Sample size	Number of SNPs	Population	Consortium	Sex	Year
Particulate matter(PM)	PM_2.5_ um	ukb-b-10817	423,796	9,851,867	European	MRC-IEU	Males and females	2018
	PM_2.5–10_ um	ukb-b-12963	423,796	9,851,867	European	MRC-IEU	Males and females	2018
	PM_10_ um	ukb-b-589	455,314	9,851,867	European	MRC-IEU	Males and females	2018
Nitrogen dioxide		ukb-b-2618	456,380	9,851,867	European	MRC-IEU	Males and females	2018
Nitrogen oxides		ukb-b-12417	456,380	9,851,867	European	MRC-IEU	Males and females	2018

The latest data utilized for outcome analysis in this MR study was obtained from FinnGen, an extensive research collaboration between public and private sectors. This initiative merges imputed genetic data from both newly collected and existing samples within Finnish biobanks with electronic health records from Finland’s Health Registry, aiming to uncover new genetic insights into various diseases (29, 30). The dataset for allergic rhinitis, accessible via FinnGen’s public dataset, included 11,009 cases and 359,149 controls, all of European descent. Similarly, the asthma dataset, available at FinnGen’s repository, comprised 42,163 cases and 202,399 controls, also of European ancestry.

### 2.3 Selection of instrumental variables

Figure 1B outlines the initial step in our methodology, where we identified SNPs significantly linked to the gut microbiota to serve as instrumental variables (IVs) in our study. We applied a dual-tiered approach for the selection of these IVs. At the outset, our criteria demanded that SNPs must reach a level of genome-wide significance (*p* < 5 × 10–^8^) to qualify as IVs. This strict standard, however, resulted in the inclusion of only PM10 as IVs, narrowing the breadth of our investigation. In pursuit of a broader analysis that could delve into the intricate connections between pancreatitis, air pollution, and achieve more encompassing outcomes, we opted for a slightly relaxed selection threshold of *p* < 5 × 10–^6^ for the SNP IVs.

During the IV extraction phase, we meticulously screened out SNPs affected by linkage disequilibrium (LD), adhering to parameters of r2 < 0.001 and kb > 10,000 (31), to maintain the integrity of our instrumental variables. In instances where the initially selected SNP was not present in the GWAS findings, we employed a proxy SNP with a high linkage disequilibrium (r^2^ > 0.8) as a substitute (32). This step ensured the consistency of the allele’s impact on both the exposure and the outcome by excluding palindromic SNPs.

Conclusively, we assessed each chosen SNP for its R^2^ (33) and F-statistic (34), following established protocols. The F-statistic helped us gage the potential for weak instrumental variable bias, whereas R2 quantified the variability in iron status attributed to the SNP. Our selection criteria ensured that each SNP presented an F-statistic above 10, signifying their robust predictive power regarding the exposure (35). Detailed SNP metrics, including the corresponding R^2^ and F-statistic, are cataloged in the Supplementary Table.

### 2.4 Mendelian randomization analysis

In our study, we utilized the IVW methodology to investigate the causal relationship between exposure to air pollution and the development of allergic rhinitis and asthma. This approach employs the Wald ratio to derive an estimate of the causal effect from each genetic instrumental variable (IV) individually, followed by aggregating these individual estimates into a comprehensive causal effect analysis using a fixed-effect model meta-analysis. This process is designed to yield a robust and reliable estimate of the causal effect, establishing it as a cornerstone technique in the field of MR studies (36). To ensure the validity and stability of our findings, our analysis was further augmented with several Supplementary methods, including weighted mode, weighted median simple mode, weighted median, and MR-Egger regression (37). The MR-Egger regression method was particularly instrumental in assessing the presence and influence of pleiotropy among the IVs. A non-significant MR-Egger intercept (*p* > 0.05) or one that is close to zero suggests that the impact of pleiotropy on the IVs is minimal, thereby reinforcing the integrity of our causal inferences (38). Additionally, we employed Cochran’s Q test to assess heterogeneity across the IVs within the IVW framework, with a *p*-value greater than 0.05 indicating a lack of significant heterogeneity (39). The MR-PRESSO technique was also utilized to identify and correct for outliers among the SNPs, ensuring that our causal estimates remained accurate by excluding these outliers from the analysis (40). Moreover, a leave-one-out sensitivity analysis was conducted, sequentially excluding each SNP to determine if any specific SNP had a disproportionate influence on the overall results. This step was crucial for reducing potential errors in screening IVs and enhancing the reliability of our causal estimates (41). Through these meticulous and comprehensive analytical procedures, our study aimed to provide a more accurate and reliable understanding of the causal links between air pollution and respiratory allergic conditions.

### 2.5 Statistical analysis

The analyses in this investigation were executed utilizing the “TwoSampleMR” (32) and “MR-PRESSO” (40) tools available within the R Foundation software, version 4.2.2. We established a *p*-value of less than 0.05 as the benchmark for determining statistical significance in our evidence.

## 3 Results

### 3.1 Genetic IVs extraction of air pollution from the allergic rhinitis and asthma GWAS dataset

In conducting our research, we carefully removed instrumental variables with linkage disequilibrium from the genome-wide association study (GWAS) dataset related to allergic rhinitis. This process led us to identify a set of unique SNPs significantly associated with specific air pollutants, with the level of significance set at *p* < 5 × 10–^6^. Our findings included 8 SNPs closely linked to PM2.5 concentration, 23 to PM2.5–10, 231 to PM10, 98 to nitrogen dioxide, and 72 to nitrogen oxides, showcasing a clear genetic predisposition to these environmental exposures.

A similar approach applied to the asthma GWAS dataset revealed a distinct pattern of associations: 8 SNPs were identified for PM2.5, 23 for PM2.5–10, 22 for PM10, 5 for nitrogen dioxide, and 8 for nitrogen oxides, indicating a robust genetic basis for susceptibility to these pollutants.

To further ensure the robustness of our instrumental variables against the potential dilution of weak instruments, we employed the F statistic to assess the strength of the correlation between each IV and its corresponding exposure. The analysis confirmed the absence of significant weak instrumental variables among the SNPs selected (all *F* > 10), thereby reinforcing the reliability of our causal estimations. The detailed attributes of these instrumental variables, including their respective exposures and the F statistic values, are meticulously cataloged in the Supplementary Table, providing a comprehensive overview of the genetic determinants influencing susceptibility to air pollution and its impact on allergic rhinitis and asthma.

### 3.2 Mendelian randomization analysis

#### 3.2.1 Allergic rhinitis

**Figure 2:**
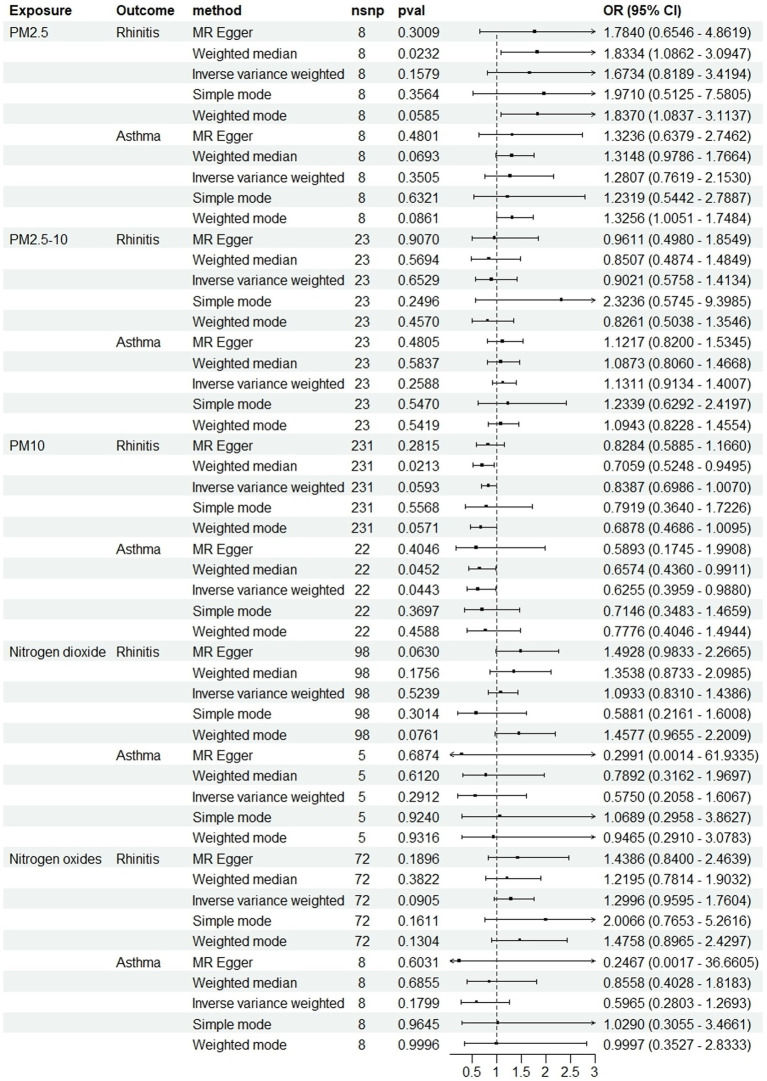
Causal linkage of air pollution exposure with allergic rhinitis and asthma outcomes. nSNP, SNP count; OR, odds ratio; CI, confidence interval.

**Figure 3:**
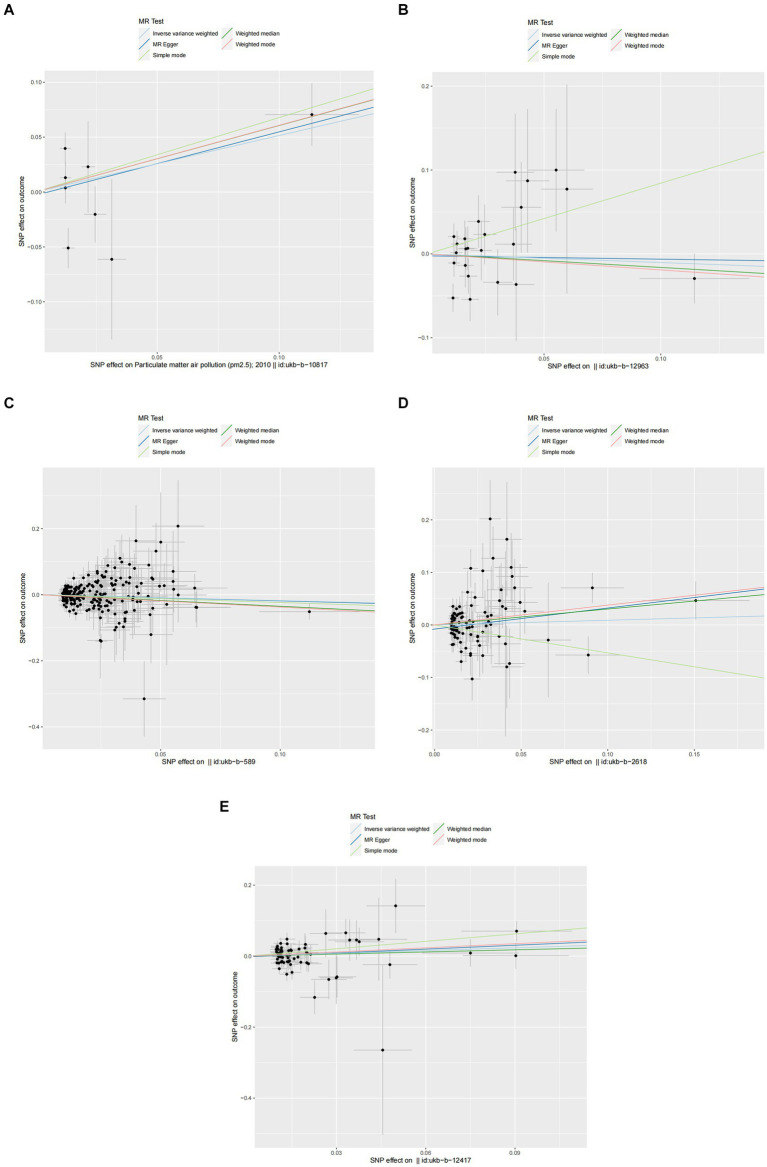
Scatter plots of SNPs associated with air pollution and allergic rhinitis. Each black point representing each SNP on the exposure (horizontal-axis) and on the outcome (vertical-axis) is plotted with error bars corresponding to each standard error (SE). The Mendelian randomization (MR) regression slopes of the lines represent the causal estimates using five approaches (inverse-variance weighted (IVW), MR-Egger, weighted median, simple mode, and weighted mode). **(A)** PM2.5. **(B)** PM2.5–10. **(C)** PM10. **(D)** Nitrogen dioxide. **(E)** Nitrogen oxides.

**Figure 4:**
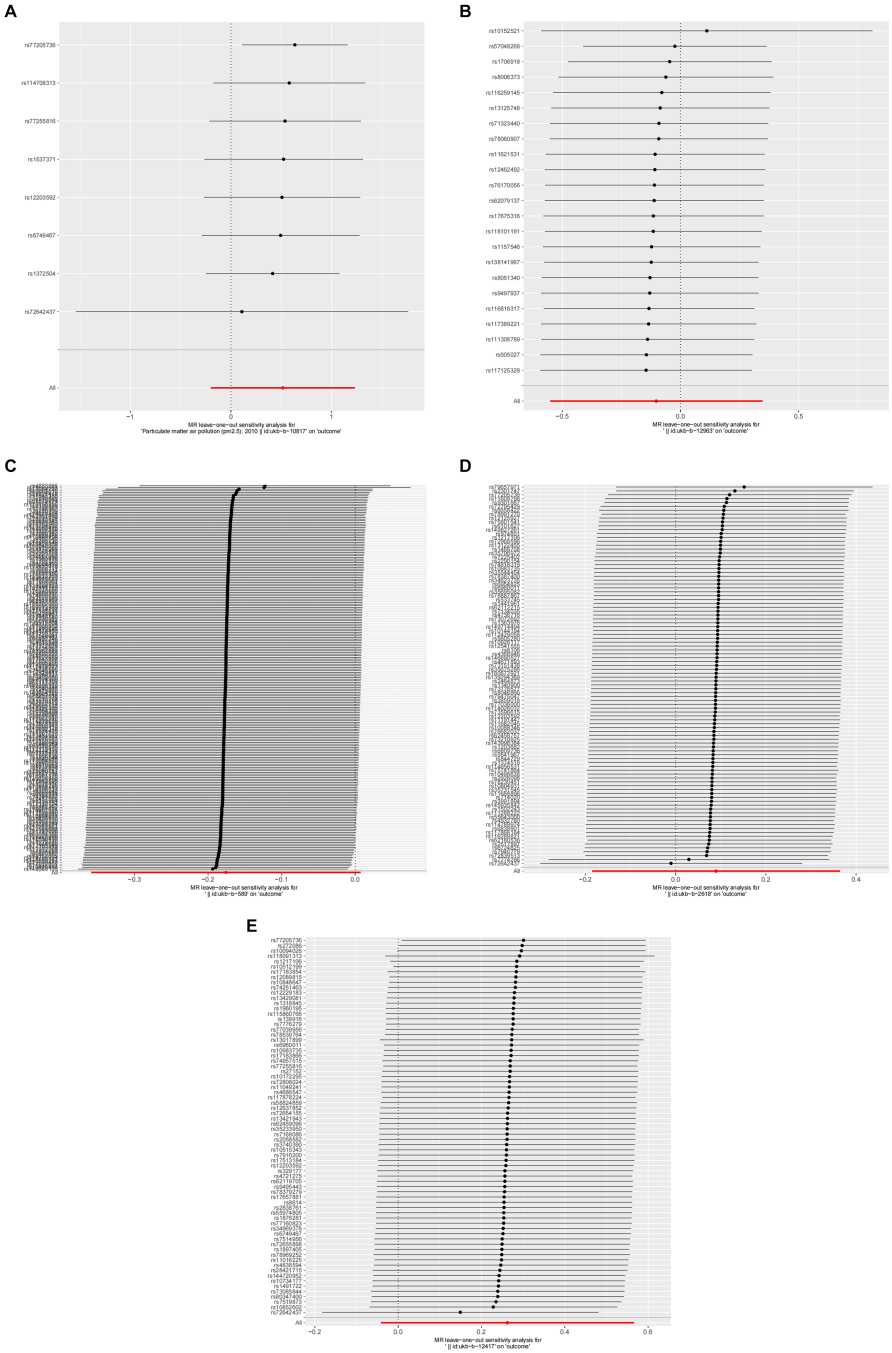
Forest plots of Leave-one-out analyses for causal SNP effect of air pollution on allergic rhinitis. The error bars indicate the 95% confidence interval (CI). **(A)** PM2.5; **(B)** PM2.5–10; **(C)** PM10; **(D)** Nitrogen dioxide; **(E)** Nitrogen oxides.

In the primary analysis conducted using the IVW method, our Mendelian Randomization study revealed the following ORs and 95% CIs for the respective exposures: For PM2.5, (OR = 1.673, 95% CI:0.819–3.419, *p* = 0.158); for PM2.5–10, (OR = 0.902, 95% CI:0.576–1.413, *p* = 0.653); PM10(OR = 0.839, 95% CI:0.699–1.007, *p* = 0.059); for nitrogen dioxide, (OR = 1.093, 95% CI: 0.831–1.439, *p* = 0.524); and for nitrogen oxides, (OR = 1.300, 95% CI:0.959–1.760, *p* = 0.090; as depicted in Figure 2). These results indicated that there was no statistically significant evidence to support a causal link between the exposures to PM2.5, PM2.5–10, PM10, nitrogen dioxide, and nitrogen oxides and the risk of developing allergic rhinitis, as illustrated in Figures 2–4.

#### 3.2.2 Asthma

**Figure 5:**
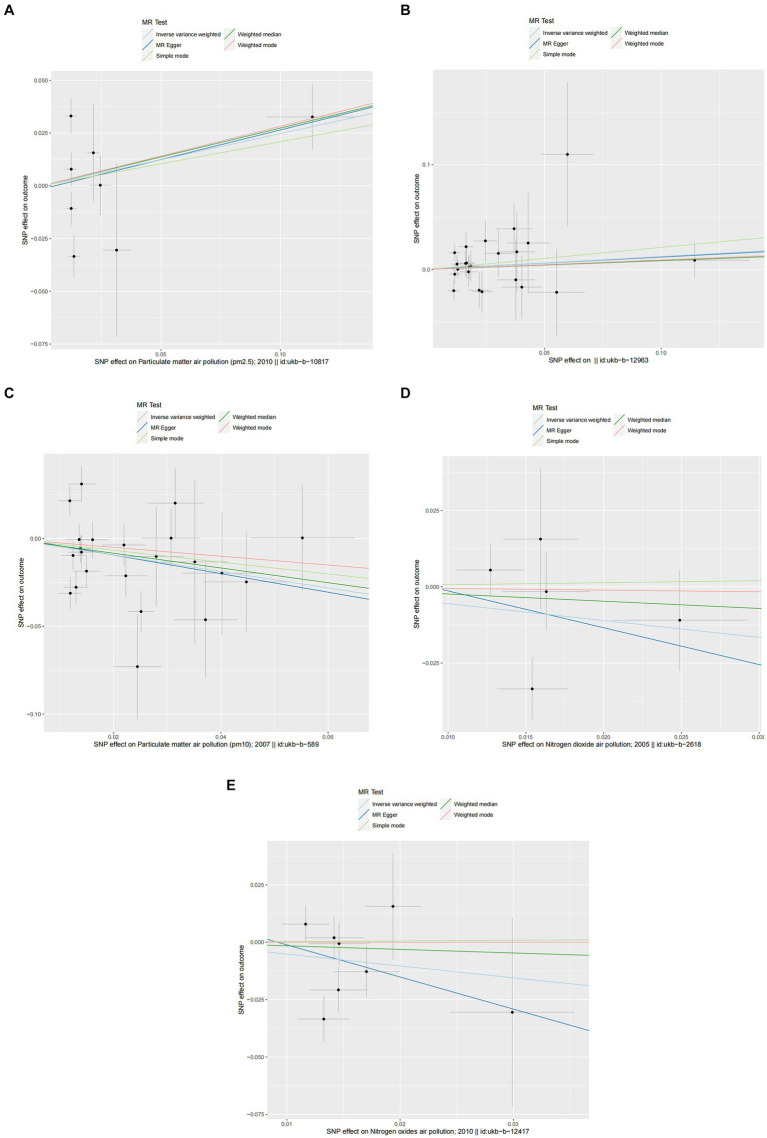
Scatter plots of SNPs associated with air pollution and asthma. Each black point representing each SNP on the exposure (horizontal-axis) and on the outcome (vertical-axis) is plotted with error bars corresponding to each standard error (SE). The Mendelian randomization (MR) regression slopes of the lines represent the causal estimates using five approaches (inverse-variance weighted (IVW), MR-Egger, weighted median, simple mode, and weighted mode). **(A)** PM2.5. **(B)** PM2.5–10. **(C)** PM10. **(D)** Nitrogen dioxide. **(E)** Nitrogen oxides.

**Figure 6:**
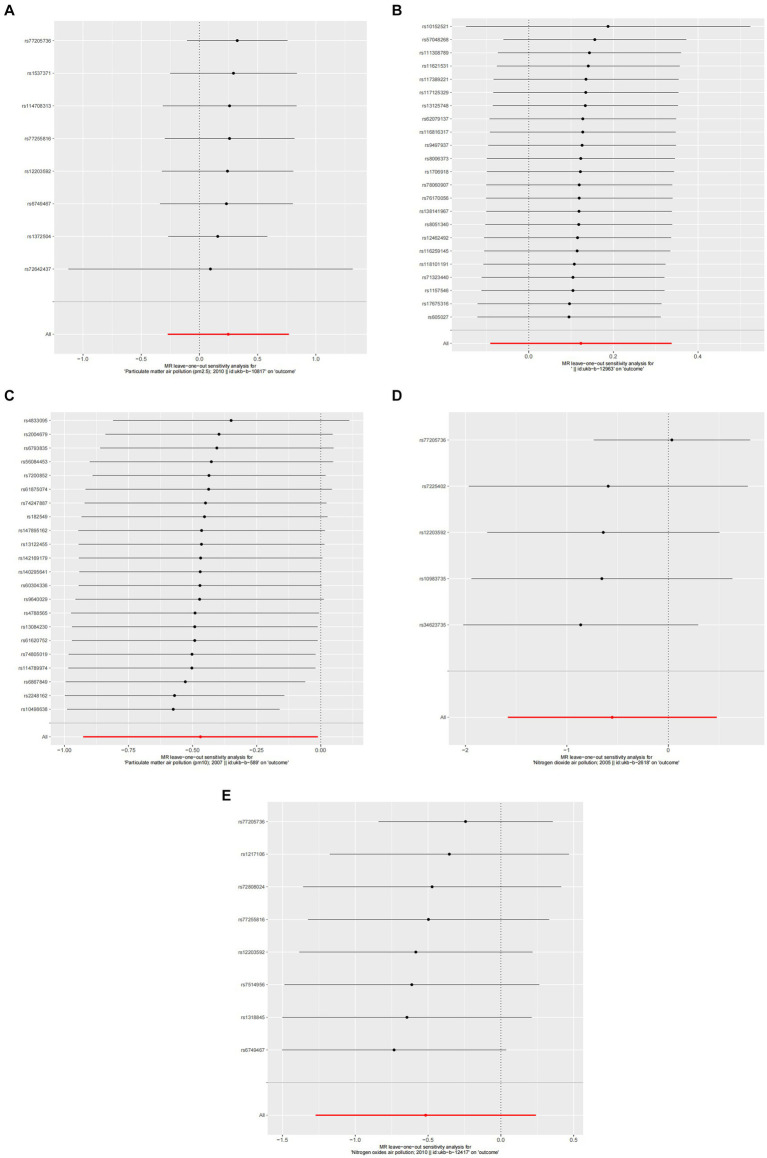
Forest plots of Leave-one-out analyses for causal SNP effect of air pollution on asthma. The error bars indicate the 95% confidence interval (CI). **(A)** PM2.5; **(B)** PM2.5–10; **(C)** PM10; **(D)** Nitrogen dioxide; **(E)** Nitrogen oxides.

In the core analysis utilizing the IVW method, our MR investigation revealed the following data on the impact of different air pollutants: For PM2.5 exposure, (OR = 1.281, 95% CI:0.762–2.153, *p* = 0.350); for PM2.5–10 exposure, (OR = 1.131, 95% CI:0.913–1.401, *p* = 0.259); for PM10 exposure, (OR = 0.625, 95% CI: 0.396–0.988, *p* = 0.044); for nitrogen dioxide exposure, (OR = 0.575, 95% CI:0.206–1.607, *p* = 0.291); and for nitrogen oxides exposure, (OR = 0.596, 95% CI:0.280–1.269, *p* = 0.180; referenced in Figure 2). These findings pinpointed a significant causal link specifically between PM10 exposure and an increased risk of asthma, highlighting that asthma risk escalates with each standard error increment in PM10 levels, as illustrated in Figures 2, 5, 6. Conversely, the study found no statistically significant causal connections between exposures to PM2.5, PM2.5–10, nitrogen dioxide, nitrogen oxides, and the incidence of asthma, as detailed in Figures 2, 5, 6.

### 3.3 Pleiotropy and heterogeneity analysis

The MR-Egger intercept test results for allergic rhinitis indicated the absence of pleiotropy (*p* > 0.05, as detailed in Table 2), suggesting that the SNPs associated with the exposures under investigation do not directly contribute to allergic rhinitis, independent of their exposure levels. The analysis also detected heterogeneity in the data for various pollutants (PM_2.5_: *p* = 0.089, Q = 18.789; PM_2.5–10_: *p* = 0.118, Q = 30.015; PM_10_: *p* = 0.088, Q = 283.967; nitrogen dioxide: *p* < 0.001, Q = 154.568; nitrogen oxides: *p* = 0.232, Q = 96.648, as seen in Table 2).

**Table 2:** Assessment of air pollution genetic determinants’ pleiotropy and heterogeneity in GWAS, targeting allergic rhinitis and asthma.

Outcomes	Exposures	Pleiotropy test			Heterogeneity tests		
		egger_intercept	se	pval	Q	Q_df	Q_pval
Allergic rhinitis	PM_2.5_	−0.003	0.016	0.852	18.789	7.000	0.089
	PM_2.5–10_	−0.002	0.009	0.795	30.015	22.000	0.118
	PM_10_	0.000	0.003	0.933	283.967	230.000	0.088
	Nitrogen dioxide	−0.008	0.004	0.058	154.568	97.000	0.000
	Nitrogen oxides	−0.002	0.005	0.655	96.648	71.000	0.232
Asthma	PM_2.5_	−0.002	0.011	0.895	32.270	7.000	0.585
	PM_2.5–10_	0.000	0.004	0.943	21.938	22.000	0.464
	PM_10_	0.001	0.011	0.918	58.387	21.000	0.060
	Nitrogen dioxide	0.011	0.043	0.821	9.717	4.000	0.055
	Nitrogen oxides	0.013	0.037	0.738	15.106	7.000	0.447

PM, Particulate matter; SE, standard error. Q_pval: *p* value of Q test from IVW method; pval, *p*-value; Q, Cochran Q statistics; SNPs, single nucleotide polymorphisms; IVW, the inverse variance.

Similarly, for asthma, the MR-Egger intercept test results showed no signs of pleiotropy (*p* > 0.05, referenced in Table 2), indicating that the SNPs linked to the pollutants studied do not have a direct contribution to asthma, irrespective of their exposure levels. Heterogeneity was observed across the pollutants (PM_2.5_: *p* = 0.585, Q = 32.270; PM_2.5–10_: *p* = 0.464, Q = 21.938; PM_10_: *p* = 0.060, Q = 58.387; nitrogen dioxide: *p* = 0.055, Q = 9.717; nitrogen oxides: *p* = 0.447, Q = 15.106, as reported in Table 2).

### 3.4 Sensitivity analysis

A detailed sensitivity analysis was conducted on the MR study results. In the case of AR, scrutinizing the influence of each individual SNP across all examined exposures demonstrated minimal effect on the derived outcomes. This finding suggests that the removal of any single SNP did not substantially alter the computed overall causal effect estimates, as depicted in Figure 4. Regarding asthma, the leave-one-out sensitivity analysis, particularly focusing on the effect of PM10, revealed that the instrumental variable lines consistently appeared to the left of the zero mark. Importantly, the omission of any single SNP did not significantly change the results, as illustrated in Figure 6. These stable MR results underscore PM10 as a contributing risk factor for asthma. Furthermore, the analysis concerning other exposures similarly showed that the exclusion of each SNP, one at a time, had negligible effects on the outcomes. This consistency further reinforces that no individual SNP significantly influences the overall causal effect estimation, as shown in Figure 6.

## 4 Discussion

In our MR investigation, we explored the potential causal links between exposure to various forms of air pollution (including particulate matter sizes PM2.5 and PM2.5–10, as well as PM10, nitrogen dioxide, and nitrogen oxides) and respiratory conditions such as allergic rhinitis and asthma. The analysis did not demonstrate a causal connection between the specified air pollutants and the incidence of allergic rhinitis. Our analysis, employing the IVW approach, indicated that elevated levels of PM10 are linked to a heightened asthma risk within a European demographic. Specifically, an increase in PM10 by each standard deviation was associated with a rise in asthma risk, based on the selection of instrumental variables using a *p*-value threshold of less than 5 × 10^−6^ (OR = 0.625, 95% CI = 0.396–0.988). The IVW model presupposes the validity of all genetic instruments and the absence of horizontal pleiotropy. In essence, our findings reveal a significant causal connection between PM10 levels and the risk of developing asthma. Conversely, we observed no causal link between other pollutants and allergic rhinitis within the European context.

The investigation into the association between allergic respiratory ailments, namely asthma and allergic rhinitis, with air pollution, has been a focal point of scientific inquiry. The principle of “one airway-one disease” underscores a unified inflammatory process in both upper and lower airways, manifesting as allergic rhinitis and asthma, respectively. These disorders not only emerge from common triggers, displaying concurrent prevalence and benefiting from similar treatment strategies due to analogous etiological factors and inflammatory cell profiles, but also exhibit a nuanced relationship with air pollution (42). Our analysis diverges from the direct causal linkage for pollutants such as PM2.5–10, PM2.5, nitrogen dioxide, and nitrogen oxides with these respiratory conditions. Nonetheless, it aligns with existing epidemiological research pinpointing PM10 as a significant asthma risk factor. Illustratively, research conducted by Chuansha Wu et al. underscores the pronounced risk of childhood asthma associated with early-life exposure to PM10 in a cohort of children aged 3 to 6 years across several Chinese cities (43). Additional studies, including one by Betancourt and a Korean study focusing on PM10 exposure during pregnancy linked to asthma in offspring among 1,572 mother-infant pairs (44, 45), further validate PM10’s detrimental effects. A study involving a Southern Taiwanese adult population also found that individuals exposed to elevated PM10 levels experienced more severe asthma symptoms (46). In light of the scarcity of research on the genetic mechanisms underpinning the link between air pollution and asthma susceptibility, our study employed a two-sample Mendelian randomization technique. This approach unveiled a significant genetic causal relationship between PM10 exposure and an increased risk of developing asthma, thereby enriching the discourse on the interplay between air pollutants and asthma.

The pathways through which PM10 elevates asthma risk remain largely elusive. Research conducted by Matteo Bonato et al. revealed that acute exposure to elevated PM10 levels correlates with diminished IFN-β expression in airway epithelial cells, potentially facilitating heightened viral replication (47). Particulate matter (PM10) has a noticeable impact on asthma, but particles with a size of 2.5 or 2.5–10 not affected it. Animal research provided strong evidence on this issue. Asthmatic mice were studied to determine the effects of PM2.5 and PM10 on Th17 cell development (48). Conclusions Asthmatic mice exposed to PM10 exhibited a dramatic increase in lung bronchiole remodeling and inflammatory cell infiltration compared to those exposed to PM2.5, according to the study. Additionally, there was a notable rise in the release of the cytokines IL-17A, IL-21, IL-22, and IL-23 by Th17 cells. As a result, when asthma attacks first appear, PM10 is believed to have a more potent pro-inflammatory impact than PM2.5. In a separate study, Huang (49) sought to determine how healthy BALB/c mice’s immune systems responded to different sizes of particulate matter (PM10), specifically PM2.5, PM1, and PM0.1 samples. An asthma model in mice was used to assess the impact of PM on the sensitization phase. Mice given high doses of PM2.5 and medium or high doses of PM10 had considerably higher neutrophil percentages in blood-borne pathogen load (BLL). Additionally, when comparing the BALF samples of mice exposed to high doses of PM10 to those of the control and other treatment groups, higher amounts of TNF-α, IFN-γ, IL-5, IL-13, IL-17A, and IL-6 were discovered. Additionally, BALF analysis demonstrated that high dosages of PM10 alone raised inflammatory cytokine expression (IL-5 and IL-13), eosinophil counts, and percentages. No influence on eosinophilic inflammation was found in extensive examination of individual PM2.5, PM1, and PM0.1 samples. When comparing mice treated with high doses of PM10 to those in the control and other treatment groups, Huang found that, in BALF, neutrophils and expression levels of inflammatory cytokines (TNF-α, IFN-γ, and IL-6) were considerably elevated. In a similar vein, an earlier research study that examined healthy mice exposed to PM from six US cities at levels of 25 μg and 100 μg found that coarse PM caused greater lung inflammation than fine or ultrafine PM (50). Coarse PM and PM2.5–1 were similarly determined by Happo et al. (51) to be far more inflammatory than PM1. Coarse PM caused higher lung inflammation than fine and ultrafine PM when comparing mass in a comparable investigation that examined size-fractioned PM toxicity in samples taken close to and far from metropolitan roadways (52). The highest level of airway inflammation observed in PM10 may be due to chemical contents attached to PM10 (53), more biogenic components with high endotoxin contents (51), or enhanced metal constituents in PM10 (54). This is just one of several potential reasons why complex urban PM can have a wide variety of mixed effects.

While there is a substantial amount of data linking PM10 to asthma, current studies investigating the impact of air pollution on the start of AR in children (55) and adults (56) have shown conflicting results. A study in France involving 36,397 patients with AR found that air pollution did not affect the severity of rhinitis (57). Conversely, air pollutants such as O3, PM2.5, PM10, SO2, and NO2 were found to have a substantial impact on AR, according to a recent study in China that involved 12,868 instances of AR outpatients (58). A other study from 2023 found that there was a correlation between short-term exposure to PM2.5, PM10, SO2, NO2, and CO with an increase in AR clinic visits (59). This contentious matter can be explained by two primary factors. Firstly, the risk of AR is increased when allergens (pollen, fungal spores, etc.) mix with air pollutants (PM10, nitrogen oxides, etc.). According to reports, air pollutants not only affect the respiratory system directly, but they also interact with plants and fungi, making pollen (from plants like ragweed or cypress) and fungal spores more allergenic (60). Ragweed, for instance, developed more quickly, flowered earlier, and produced more pollen when grown in an urban environment with high CO2 concentrations as compared to ragweed grown in a rural area (61). New evidence suggests that contaminants can indirectly trigger allergen release by damaging cells directly (12). The bioactive chemicals included in pollen grains and fungal spores have the potential to cause inflammation and allergies (62, 63). Results may vary depending on the location due to differences in air pollution and allergens. Another possible reason could be the discrepancy in the measured levels of air contaminants. Data on air pollutants, including daily average concentrations of PM2.5, PM10, SO2, NO2, CO, and O3-8h (max), were sourced from the local Municipal Ecological Environmental Bureau in 12,868 cases study (58). It goes without saying that there is no one-size-fits-all degree of contamination exposure. Alternatively, in the Changsha study (55), the data on air pollutants were measured at monitoring stations using the standard methods established by the State Environmental Protection Agency of China. The researchers then estimated individual exposure to air pollution, with the concentrations of air pollutants at kindergartens being used to calculate the exposure of the children. This method is divided into two steps (55): first, calculate the daily average concentration for each kindergarten based on the daily concentrations from the four nearest monitoring stations; second, compute the monthly average concentration of air pollutants for each kindergarten as the daily average concentration for each month.

Studies have reported several approaches about how Compute the individual level of air pollutants. The use of measuring instruments and equipment is prevalent because it provides more precise results. To achieve the personal real-time 24-h PM2.5 exposure estimates in an APACR Study (64), researchers utilized a PM2.5 size-selective intake operated by a 1.5 L/min pump in conjunction with a personal DataRam (pDR, model 1,200, Thermo Scientific, Boston MA). Also, optical PM2.5 sensors (Zefan Technol., China) were used for real-time microenvironment air pollution measurement (65). In order to regulate the airflow rate through the PM measurement channel, each monitor is outfitted with a steady-speed fan and a laser scattering sensor (Plantower PMS7003, Beijing, China). Every home that participated in the study had a real-time PM2.5 monitor installed in the kitchen, living room, and bedroom. Other ways of estimating populations from individuals have also been documented. Data on particulate matter 2.5, particulate matter 10, nitric oxide, sulfur dioxide, and CO are collected hourly from 25 monitoring stations, one for each district, by the Seoul Research Institute of Public Health and Environment. We calculated the yearly air pollutants for everyone included by combining the participants’ most recent updated addresses with the average pollution level recorded at the monitoring sites (66). However, a greater number of confounding factors make it impossible to use either population-to-individual estimation or equipment data of air contaminants (67, 68). Our study does not establish a causal relationship between air pollution and allergic rhinitis. Investigating the potential correlation between an exposure and its subsequent effects is crucial to MR research, which relies on genetic data. Similar to how randomized controlled trials (RCTs) assign participants to either a treatment or control group, MR studies “randomize” genes based on one or more alleles that affect risk factors to see if there is a difference in disease risk between carriers and non-carriers. Genetic variants are inherited and do not change during a person’s lifetime, in contrast to the usual methods of observational studies that rely on questionnaires, biochemical markers, or imaging to determine exposure. That is why MR-derived associations are immune to confounding variables and causal inversion. As a result, our study provides a means to eliminate genetic confounding factors. This is a benefit of MR studies and a difference from earlier studies.

The study employs a MR framework, presenting distinct strengths. Initially, it utilizes genetic variations as markers for exposure levels to air pollution, aligning with Mendel’s principles of genetic segregation and independent assortment. This method significantly enhances the credibility of establishing a causal link between exposure to air pollutants and the incidence of asthma. Additionally, the genetic approach inherently eliminates the possibility of reverse causation, as the genetic predispositions exist prior to disease development. Furthermore, the study capitalizes on data aggregated from comprehensive GWAS, which encompass a vast array of samples, thereby reinforcing the statistical foundation of the findings. The consideration of participants primarily of European descent further mitigates the risk of confounding effects due to population stratification, ensuring a more focused analysis. This research effectively fills a critical gap in the existing body of epidemiological studies, offering a refined theoretical and practical framework to combat the health risks associated with air pollution.

Our investigation encounters certain limitations. Initially, to enhance the applicability of our findings, it is essential to conduct further studies involving populations from different countries. This necessity stems from the reliance on a GWAS dataset of European origin for the MR analyses, which may not reflect the associations in individuals from diverse ancestries. Secondly, the scarcity of SNPs meeting the genome-wide significance threshold of 5 × 10^−8^ led us to adopt a less stringent significance level of 5 × 10^−6^ for our results. Expanding the sample size could provide more robust support for our conclusions. Given the reliance on summary statistics from a MR study for our analysis, the findings suggesting a causal link between PM10 exposure and increased asthma risk remain provisional. It is imperative to conduct more comprehensive research to uncover the specific pathways through which PM10 influences the likelihood of developing asthma.

## 5 Conclusion

Our research contributes genetic evidence supporting the notion that heightened PM10 exposure correlates with an increased asthma risk. Asthma is a condition known for its progressive nature, tends to deteriorate over time; however, its adverse impacts are reversible, manageable, and preventable. Hence, this discovery holds significant implications for public health, highlighting the detrimental effects of PM10 on the well-being of European populations. It underscores the necessity for policymakers and the general public to recognize the critical need for air quality improvements. Furthermore, the findings lay a groundwork for governments and environmental agencies to enact stricter air quality regulations and initiatives aimed at curbing PM10 emissions. Moreover, this study paves the way for further investigations into the biological underpinnings, potential confounding variables, and additional environmental contributors that influence the delicate balance between PM10 exposure and asthma risk. Such research endeavors will advance our comprehension and clarification of the intricate causal dynamics at play.

## Data availability statement

The original contributions presented in the study are included in the article/Supplementary material, further inquiries can be directed to the corresponding author.

## Author contributions

JZ: Writing – original draft, Methodology, Data curation. WL: Writing – review & editing, Software, Investigation. SY: Writing – original draft, Resources, Project administration, Investigation, Formal analysis. YS: Writing – original draft, Supervision, Investigation, Data curation, Conceptualization. XL: Writing – review & editing, Writing – original draft, Supervision, Funding acquisition, Data curation.
