# Supplementary material for: Causal association between air pollution and allergic rhinitis, asthma: a Mendelian randomization study
Source: Front Public Health. 2024 Jul 15;12:1386341. doi: 10.3389/fpubh.2024.1386341 (PMC11284075; doi:10.3389/fpubh.2024.1386341)
Supplement: Supplementary file 1 [file Data_Sheet_1.docx]

Supplementary Material

# Supplementary Table 1

Genetic variants significantly associated with air pollution exposure as instrumental variables and allergic rhinitis as outcome.

| **exposures** | **SNP** | **effect_allele.exposure** | **other_allele.exposure** | **beta.exposure** | **se.exposure** | **pval.exposure** | **eaf.exposure** | **R2** | **f** |
| --- | --- | --- | --- | --- | --- | --- | --- | --- | --- |
| PM2.5 | rs114708313 | T | A | 0.024558 | 0.00447797 | 4.20E-08 | 0.06585 | 7.10E-05 | 30.07610422 |
|  | rs12203592 | T | C | 0.0216661 | 0.00259111 | 6.20E-17 | 0.212894 | 0.000164953 | 69.91779705 |
|  | rs1372504 | A | G | 0.0122914 | 0.00221931 | 3.10E-08 | 0.374311 | 7.24E-05 | 30.67359752 |
|  | rs1537371 | A | C | 0.0123705 | 0.00214859 | 8.50E-09 | 0.500143 | 7.82E-05 | 33.1486127 |
|  | rs6749467 | A | G | -0.0123919 | 0.00218282 | 1.40E-08 | 0.465814 | 7.60E-05 | 32.22833743 |
|  | rs72642437 | T | C | 0.113396 | 0.019135 | 3.10E-09 | 0.003862 | 8.29E-05 | 35.11854286 |
|  | rs77205736 | T | C | 0.0135219 | 0.00241312 | 2.10E-08 | 0.273909 | 7.41E-05 | 31.39898085 |
|  | rs77255816 | T | C | 0.0313937 | 0.00572778 | 4.20E-08 | 0.036507 | 7.09E-05 | 30.04071746 |
| PM2.5-10 | rs10152521 | C | T | 0.114394 | 0.0234312 | 1.00E-06 | 0.002527 | 5.62E-05 | 23.83501232 |
|  | rs111308789 | A | T | -0.0219385 | 0.00472875 | 3.50E-06 | 0.056635 | 5.08E-05 | 21.52380765 |
|  | rs1157546 | C | T | -0.0246035 | 0.00490067 | 5.20E-07 | 0.053047 | 5.95E-05 | 25.20465849 |
|  | rs11621531 | A | G | -0.0231281 | 0.00448732 | 2.50E-07 | 0.063928 | 6.27E-05 | 26.56463061 |
|  | rs116259145 | A | C | 0.0301641 | 0.00618506 | 1.10E-06 | 0.031996 | 5.61E-05 | 23.78432662 |
|  | rs116816317 | A | G | 0.0376491 | 0.00796504 | 2.30E-06 | 0.019771 | 5.27E-05 | 22.34247218 |
|  | rs117125329 | G | C | 0.0551672 | 0.0119924 | 4.20E-06 | 0.008295 | 4.99E-05 | 21.16155721 |
|  | rs117389221 | C | T | -0.0402216 | 0.00850122 | 2.20E-06 | 0.017253 | 5.28E-05 | 22.38484674 |
|  | rs118101191 | T | G | 0.0597911 | 0.0110633 | 6.50E-08 | 0.010103 | 6.89E-05 | 29.20798917 |
|  | rs12462492 | T | G | -0.0123914 | 0.00249623 | 6.90E-07 | 0.257064 | 5.81E-05 | 24.64163423 |
|  | rs13125748 | A | C | 0.0114526 | 0.0025016 | 4.70E-06 | 0.257878 | 4.95E-05 | 20.95899235 |
|  | rs138141967 | T | G | -0.0429378 | 0.00915495 | 2.70E-06 | 0.01498 | 5.19E-05 | 21.99710736 |
|  | rs1706918 | A | G | 0.018349 | 0.00367669 | 6.00E-07 | 0.098932 | 5.88E-05 | 24.90627185 |
|  | rs17675316 | G | A | 0.0369053 | 0.00788044 | 2.80E-06 | 0.021631 | 5.17E-05 | 21.93181112 |
|  | rs57048268 | C | A | -0.0110199 | 0.00236534 | 3.20E-06 | 0.311303 | 5.12E-05 | 21.70531561 |
|  | rs605027 | T | C | -0.0113937 | 0.00245355 | 3.40E-06 | 0.729529 | 5.09E-05 | 21.56441521 |
|  | rs62079137 | C | T | -0.0173504 | 0.00374272 | 3.60E-06 | 0.107445 | 5.07E-05 | 21.49028909 |
|  | rs71323440 | T | C | 0.0162929 | 0.00340779 | 1.70E-06 | 0.114944 | 5.39E-05 | 22.85857147 |
|  | rs76170056 | A | C | 0.0165077 | 0.003462 | 1.90E-06 | 0.110936 | 5.36E-05 | 22.73615172 |
|  | rs78060907 | A | C | -0.0380539 | 0.00769756 | 7.70E-07 | 0.020337 | 5.77E-05 | 24.43937696 |
|  | rs8006373 | A | T | -0.0175837 | 0.00377824 | 3.30E-06 | 0.091464 | 5.11E-05 | 21.65904973 |
|  | rs8051340 | G | C | 0.0160734 | 0.00332586 | 1.30E-06 | 0.122584 | 5.51E-05 | 23.35637975 |
|  | rs9497937 | A | C | -0.0127713 | 0.00257727 | 7.20E-07 | 0.232437 | 5.79E-05 | 24.55547428 |
| PM10 | rs10498638 | C | T | 0.0140135 | 0.00253691 | 3.30E-08 | 0.188322 | 6.70E-05 | 30.51273992 |
|  | rs114789974 | A | C | -0.0552064 | 0.00963471 | 1.00E-08 | 0.010467 | 7.21E-05 | 32.83217498 |
|  | rs13084230 | T | C | -0.013562 | 0.00245992 | 3.50E-08 | 0.200319 | 6.68E-05 | 30.39510202 |
|  | rs13122455 | T | C | -0.0140039 | 0.0024643 | 1.30E-08 | 0.199995 | 7.09E-05 | 32.2930407 |
|  | rs140295641 | A | T | -0.0351352 | 0.00617205 | 1.30E-08 | 0.027357 | 7.12E-05 | 32.40589952 |
|  | rs142169179 | A | G | 0.040185 | 0.0073403 | 4.40E-08 | 0.020279 | 6.58E-05 | 29.97080566 |
|  | rs147895162 | C | T | -0.0447597 | 0.00810727 | 3.40E-08 | 0.015135 | 6.69E-05 | 30.48057482 |
|  | rs182549 | T | C | -0.0124233 | 0.00221854 | 2.10E-08 | 0.738828 | 6.89E-05 | 31.35721697 |
|  | rs2004679 | C | T | 0.0119145 | 0.00213821 | 2.50E-08 | 0.307685 | 6.82E-05 | 31.04910049 |
|  | rs2248162 | C | T | 0.0118186 | 0.00204712 | 7.80E-09 | 0.63988 | 7.32E-05 | 33.33063273 |
|  | rs4788565 | A | G | -0.0219191 | 0.00399449 | 4.10E-08 | 0.066776 | 6.61E-05 | 30.11069999 |
|  | rs4833095 | C | T | 0.0251105 | 0.00240554 | 1.70E-25 | 0.206945 | 0.00023926 | 108.964153 |
|  | rs56084453 | G | A | 0.014935 | 0.00241164 | 5.90E-10 | 0.209746 | 8.42E-05 | 38.35160901 |
|  | rs60304336 | T | G | 0.0279318 | 0.00502954 | 2.80E-08 | 0.040906 | 6.77E-05 | 30.841778 |
|  | rs61620752 | G | T | 0.0160687 | 0.00276719 | 6.40E-09 | 0.148343 | 7.41E-05 | 33.71953822 |
|  | rs61875074 | C | A | 0.0222662 | 0.00384125 | 6.80E-09 | 0.073029 | 7.38E-05 | 33.6004549 |
|  | rs6793835 | A | G | -0.0129858 | 0.00223823 | 6.60E-09 | 0.263796 | 7.39E-05 | 33.66092816 |
|  | rs6867849 | T | A | -0.0314765 | 0.00523168 | 1.80E-09 | 0.040477 | 7.95E-05 | 36.19833699 |
|  | rs7200852 | A | C | -0.0244283 | 0.00445039 | 4.00E-08 | 0.054732 | 6.62E-05 | 30.12925825 |
|  | rs74247887 | T | C | 0.037132 | 0.00588066 | 2.70E-10 | 0.028721 | 8.76E-05 | 39.8696694 |
|  | rs74805019 | C | G | -0.0307133 | 0.00548224 | 2.10E-08 | 0.033712 | 6.89E-05 | 31.38592755 |
|  | rs9640029 | T | C | -0.0138024 | 0.00197383 | 2.70E-12 | 0.478233 | 0.000107382 | 48.89763113 |
| Nitrogen dioxide | rs10983735 | A | G | 0.0163113 | 0.00280286 | 5.90E-09 | 0.154461 | 7.42E-05 | 33.86666533 |
|  | rs12203592 | T | C | 0.0159413 | 0.002408 | 3.60E-11 | 0.219436 | 9.60E-05 | 43.82607739 |
|  | rs34623735 | T | C | 0.0127201 | 0.00215704 | 3.70E-09 | 0.33449 | 7.62E-05 | 34.77464863 |
|  | rs7225402 | C | T | -0.0248919 | 0.0043153 | 8.00E-09 | 0.058308 | 7.29E-05 | 33.2730188 |
|  | rs77205736 | T | C | 0.0154062 | 0.00226879 | 1.10E-11 | 0.274855 | 0.000101026 | 46.11057747 |
| Nitrogen oxides | rs1217106 | G | A | 0.0145574 | 0.00251872 | 7.50E-09 | 0.782419 | 7.32E-05 | 33.40457466 |
|  | rs12203592 | T | C | 0.01937 | 0.00245674 | 3.20E-15 | 0.219436 | 0.000136193 | 62.16399893 |
|  | rs1318845 | C | T | -0.0141726 | 0.00259612 | 4.80E-08 | 0.200795 | 6.53E-05 | 29.80215238 |
|  | rs6749467 | A | G | -0.0116612 | 0.00209584 | 2.60E-08 | 0.46467 | 6.78E-05 | 30.95767489 |
|  | rs72808024 | C | A | -0.0170289 | 0.00290612 | 4.60E-09 | 0.148435 | 7.52E-05 | 34.33556518 |
|  | rs7514956 | C | A | -0.0146107 | 0.00265248 | 3.60E-08 | 0.186513 | 6.65E-05 | 30.3414204 |
|  | rs77205736 | T | C | 0.0132649 | 0.00231459 | 1.00E-08 | 0.274855 | 7.20E-05 | 32.84414135 |
|  | rs77255816 | T | C | 0.0299054 | 0.00547093 | 4.60E-08 | 0.036924 | 6.55E-05 | 29.87961602 |

# Supplementary Table 2

Genetic variants significantly associated with air pollution exposure as instrumental variables and asthma as outcome.

| **exposures** | **SNP** | **effect_allele.exposure** | **other_allele.exposure** | **beta.exposure** | **se.exposure** | **pval.exposure** | **eaf.exposure** | **R2** | **f** |
| --- | --- | --- | --- | --- | --- | --- | --- | --- | --- |
| PM2.5 | rs114708313 | T | A | 0.024558 | 0.00447797 | 4.20E-08 | 0.06585 | 7.10E-05 | 30.07610422 |
|  | rs12203592 | T | C | 0.0216661 | 0.00259111 | 6.20E-17 | 0.212894 | 0.000164953 | 69.91779705 |
|  | rs1372504 | A | G | 0.0122914 | 0.00221931 | 3.10E-08 | 0.374311 | 7.24E-05 | 30.67359752 |
|  | rs1537371 | A | C | 0.0123705 | 0.00214859 | 8.50E-09 | 0.500143 | 7.82E-05 | 33.1486127 |
|  | rs6749467 | A | G | -0.0123919 | 0.00218282 | 1.40E-08 | 0.465814 | 7.60E-05 | 32.22833743 |
|  | rs72642437 | T | C | 0.113396 | 0.019135 | 3.10E-09 | 0.003862 | 8.29E-05 | 35.11854286 |
|  | rs77205736 | T | C | 0.0135219 | 0.00241312 | 2.10E-08 | 0.273909 | 7.41E-05 | 31.39898085 |
|  | rs77255816 | T | C | 0.0313937 | 0.00572778 | 4.20E-08 | 0.036507 | 7.09E-05 | 30.04071746 |
| PM2.5-10 | rs10152521 | C | T | 0.114394 | 0.0234312 | 1.00E-06 | 0.002527 | 5.62E-05 | 23.83501232 |
|  | rs111308789 | A | T | -0.0219385 | 0.00472875 | 3.50E-06 | 0.056635 | 5.08E-05 | 21.52380765 |
|  | rs1157546 | C | T | -0.0246035 | 0.00490067 | 5.20E-07 | 0.053047 | 5.95E-05 | 25.20465849 |
|  | rs11621531 | A | G | -0.0231281 | 0.00448732 | 2.50E-07 | 0.063928 | 6.27E-05 | 26.56463061 |
|  | rs116259145 | A | C | 0.0301641 | 0.00618506 | 1.10E-06 | 0.031996 | 5.61E-05 | 23.78432662 |
|  | rs116816317 | A | G | 0.0376491 | 0.00796504 | 2.30E-06 | 0.019771 | 5.27E-05 | 22.34247218 |
|  | rs117125329 | G | C | 0.0551672 | 0.0119924 | 4.20E-06 | 0.008295 | 4.99E-05 | 21.16155721 |
|  | rs117389221 | C | T | -0.0402216 | 0.00850122 | 2.20E-06 | 0.017253 | 5.28E-05 | 22.38484674 |
|  | rs118101191 | T | G | 0.0597911 | 0.0110633 | 6.50E-08 | 0.010103 | 6.89E-05 | 29.20798917 |
|  | rs12462492 | T | G | -0.0123914 | 0.00249623 | 6.90E-07 | 0.257064 | 5.81E-05 | 24.64163423 |
|  | rs13125748 | A | C | 0.0114526 | 0.0025016 | 4.70E-06 | 0.257878 | 4.95E-05 | 20.95899235 |
|  | rs138141967 | T | G | -0.0429378 | 0.00915495 | 2.70E-06 | 0.01498 | 5.19E-05 | 21.99710736 |
|  | rs1706918 | A | G | 0.018349 | 0.00367669 | 6.00E-07 | 0.098932 | 5.88E-05 | 24.90627185 |
|  | rs17675316 | G | A | 0.0369053 | 0.00788044 | 2.80E-06 | 0.021631 | 5.17E-05 | 21.93181112 |
|  | rs57048268 | C | A | -0.0110199 | 0.00236534 | 3.20E-06 | 0.311303 | 5.12E-05 | 21.70531561 |
|  | rs605027 | T | C | -0.0113937 | 0.00245355 | 3.40E-06 | 0.729529 | 5.09E-05 | 21.56441521 |
|  | rs62079137 | C | T | -0.0173504 | 0.00374272 | 3.60E-06 | 0.107445 | 5.07E-05 | 21.49028909 |
|  | rs71323440 | T | C | 0.0162929 | 0.00340779 | 1.70E-06 | 0.114944 | 5.39E-05 | 22.85857147 |
|  | rs76170056 | A | C | 0.0165077 | 0.003462 | 1.90E-06 | 0.110936 | 5.36E-05 | 22.73615172 |
|  | rs78060907 | A | C | -0.0380539 | 0.00769756 | 7.70E-07 | 0.020337 | 5.77E-05 | 24.43937696 |
|  | rs8006373 | A | T | -0.0175837 | 0.00377824 | 3.30E-06 | 0.091464 | 5.11E-05 | 21.65904973 |
|  | rs8051340 | G | C | 0.0160734 | 0.00332586 | 1.30E-06 | 0.122584 | 5.51E-05 | 23.35637975 |
|  | rs9497937 | A | C | -0.0127713 | 0.00257727 | 7.20E-07 | 0.232437 | 5.79E-05 | 24.55547428 |
| PM10 | rs10498638 | C | T | 0.0140135 | 0.00253691 | 3.30E-08 | 0.188322 | 6.70E-05 | 30.51273992 |
|  | rs114789974 | A | C | -0.0552064 | 0.00963471 | 1.00E-08 | 0.010467 | 7.21E-05 | 32.83217498 |
|  | rs13084230 | T | C | -0.013562 | 0.00245992 | 3.50E-08 | 0.200319 | 6.68E-05 | 30.39510202 |
|  | rs13122455 | T | C | -0.0140039 | 0.0024643 | 1.30E-08 | 0.199995 | 7.09E-05 | 32.2930407 |
|  | rs140295641 | A | T | -0.0351352 | 0.00617205 | 1.30E-08 | 0.027357 | 7.12E-05 | 32.40589952 |
|  | rs142169179 | A | G | 0.040185 | 0.0073403 | 4.40E-08 | 0.020279 | 6.58E-05 | 29.97080566 |
|  | rs147895162 | C | T | -0.0447597 | 0.00810727 | 3.40E-08 | 0.015135 | 6.69E-05 | 30.48057482 |
|  | rs182549 | T | C | -0.0124233 | 0.00221854 | 2.10E-08 | 0.738828 | 6.89E-05 | 31.35721697 |
|  | rs2004679 | C | T | 0.0119145 | 0.00213821 | 2.50E-08 | 0.307685 | 6.82E-05 | 31.04910049 |
|  | rs2248162 | C | T | 0.0118186 | 0.00204712 | 7.80E-09 | 0.63988 | 7.32E-05 | 33.33063273 |
|  | rs4788565 | A | G | -0.0219191 | 0.00399449 | 4.10E-08 | 0.066776 | 6.61E-05 | 30.11069999 |
|  | rs4833095 | C | T | 0.0251105 | 0.00240554 | 1.70E-25 | 0.206945 | 0.00023926 | 108.964153 |
|  | rs56084453 | G | A | 0.014935 | 0.00241164 | 5.90E-10 | 0.209746 | 8.42E-05 | 38.35160901 |
|  | rs60304336 | T | G | 0.0279318 | 0.00502954 | 2.80E-08 | 0.040906 | 6.77E-05 | 30.841778 |
|  | rs61620752 | G | T | 0.0160687 | 0.00276719 | 6.40E-09 | 0.148343 | 7.41E-05 | 33.71953822 |
|  | rs61875074 | C | A | 0.0222662 | 0.00384125 | 6.80E-09 | 0.073029 | 7.38E-05 | 33.6004549 |
|  | rs6793835 | A | G | -0.0129858 | 0.00223823 | 6.60E-09 | 0.263796 | 7.39E-05 | 33.66092816 |
|  | rs6867849 | T | A | -0.0314765 | 0.00523168 | 1.80E-09 | 0.040477 | 7.95E-05 | 36.19833699 |
|  | rs7200852 | A | C | -0.0244283 | 0.00445039 | 4.00E-08 | 0.054732 | 6.62E-05 | 30.12925825 |
|  | rs74247887 | T | C | 0.037132 | 0.00588066 | 2.70E-10 | 0.028721 | 8.76E-05 | 39.8696694 |
|  | rs74805019 | C | G | -0.0307133 | 0.00548224 | 2.10E-08 | 0.033712 | 6.89E-05 | 31.38592755 |
|  | rs9640029 | T | C | -0.0138024 | 0.00197383 | 2.70E-12 | 0.478233 | 0.000107382 | 48.89763113 |
| Nitrogen dioxide | rs10983735 | A | G | 0.0163113 | 0.00280286 | 5.90E-09 | 0.154461 | 7.42E-05 | 33.86666533 |
|  | rs12203592 | T | C | 0.0159413 | 0.002408 | 3.60E-11 | 0.219436 | 9.60E-05 | 43.82607739 |
|  | rs34623735 | T | C | 0.0127201 | 0.00215704 | 3.70E-09 | 0.33449 | 7.62E-05 | 34.77464863 |
|  | rs7225402 | C | T | -0.0248919 | 0.0043153 | 8.00E-09 | 0.058308 | 7.29E-05 | 33.2730188 |
|  | rs77205736 | T | C | 0.0154062 | 0.00226879 | 1.10E-11 | 0.274855 | 0.000101026 | 46.11057747 |
| Nitrogen oxides | rs1217106 | G | A | 0.0145574 | 0.00251872 | 7.50E-09 | 0.782419 | 7.32E-05 | 33.40457466 |
|  | rs12203592 | T | C | 0.01937 | 0.00245674 | 3.20E-15 | 0.219436 | 0.000136193 | 62.16399893 |
|  | rs1318845 | C | T | -0.0141726 | 0.00259612 | 4.80E-08 | 0.200795 | 6.53E-05 | 29.80215238 |
|  | rs6749467 | A | G | -0.0116612 | 0.00209584 | 2.60E-08 | 0.46467 | 6.78E-05 | 30.95767489 |
|  | rs72808024 | C | A | -0.0170289 | 0.00290612 | 4.60E-09 | 0.148435 | 7.52E-05 | 34.33556518 |
|  | rs7514956 | C | A | -0.0146107 | 0.00265248 | 3.60E-08 | 0.186513 | 6.65E-05 | 30.3414204 |
|  | rs77205736 | T | C | 0.0132649 | 0.00231459 | 1.00E-08 | 0.274855 | 7.20E-05 | 32.84414135 |
|  | rs77255816 | T | C | 0.0299054 | 0.00547093 | 4.60E-08 | 0.036924 | 6.55E-05 | 29.87961602 |
