# Supplementary figures and images for: Causal association between air pollution and allergic rhinitis, asthma: a Mendelian randomization study
Source: Front Public Health. 2024 Jul 15;12:1386341. doi: 10.3389/fpubh.2024.1386341 (PMC11284075; doi:10.3389/fpubh.2024.1386341)

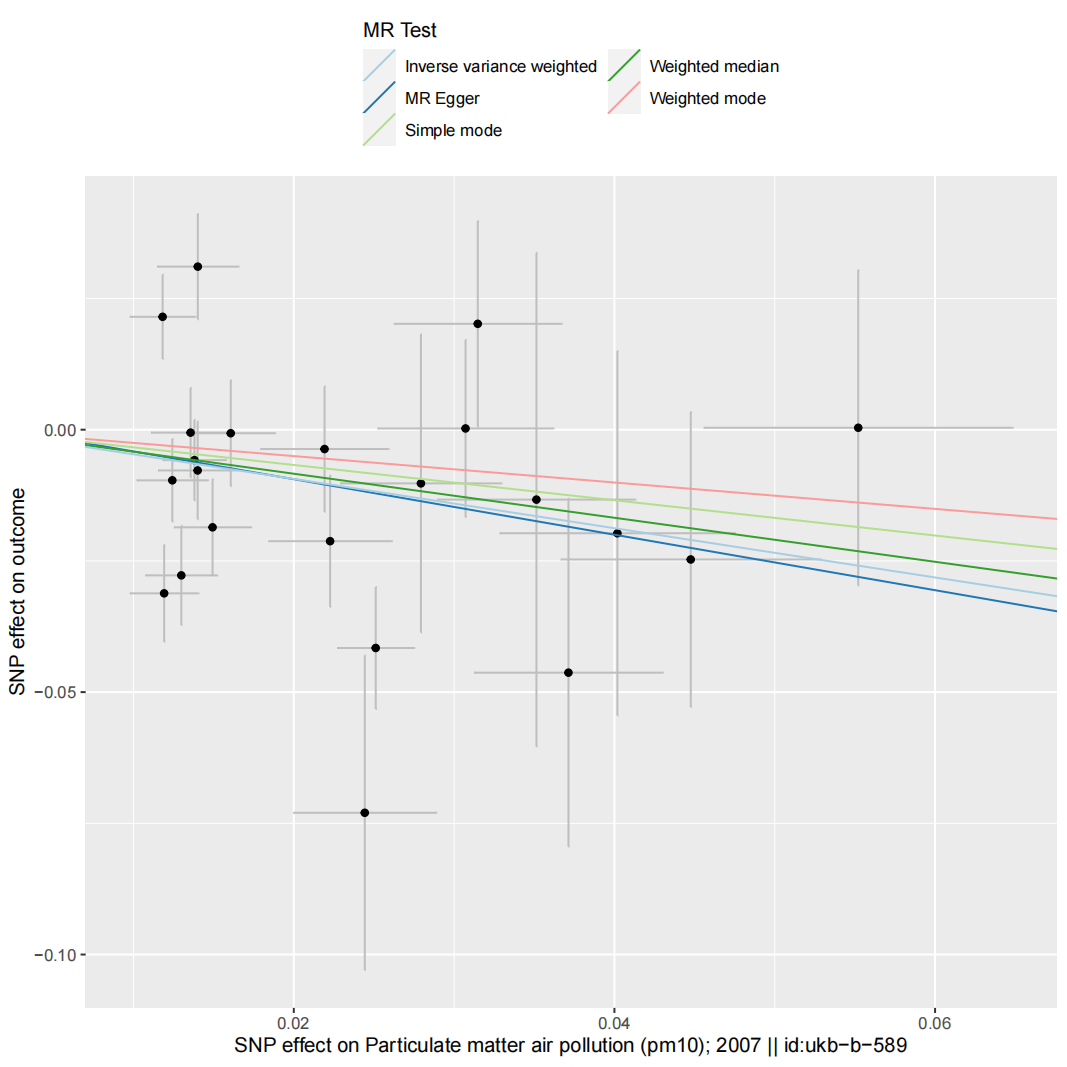

Supplement: Supplementary file 2 [file Image_1.TIF]

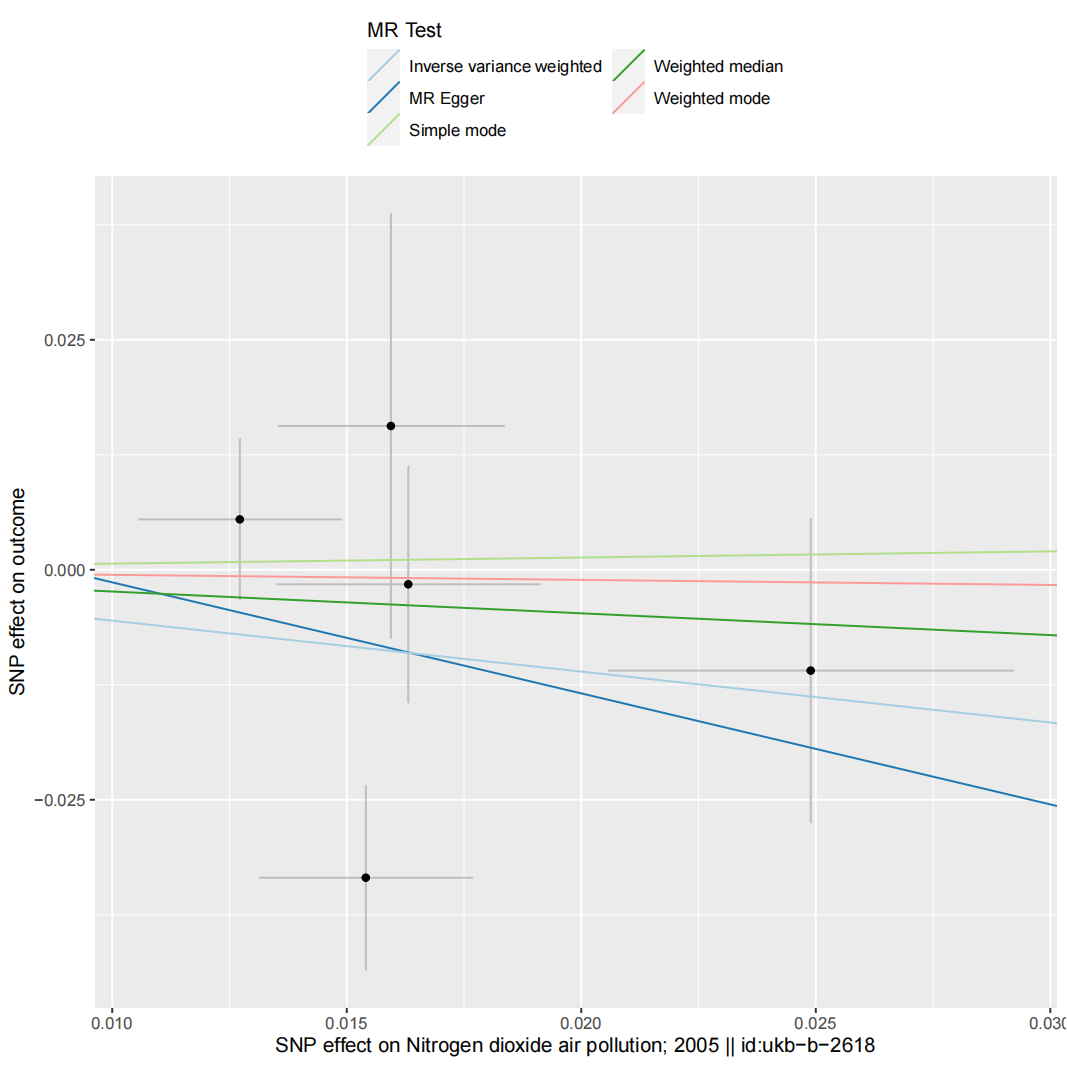

Supplement: Supplementary file 3 [file Image_2.TIF]

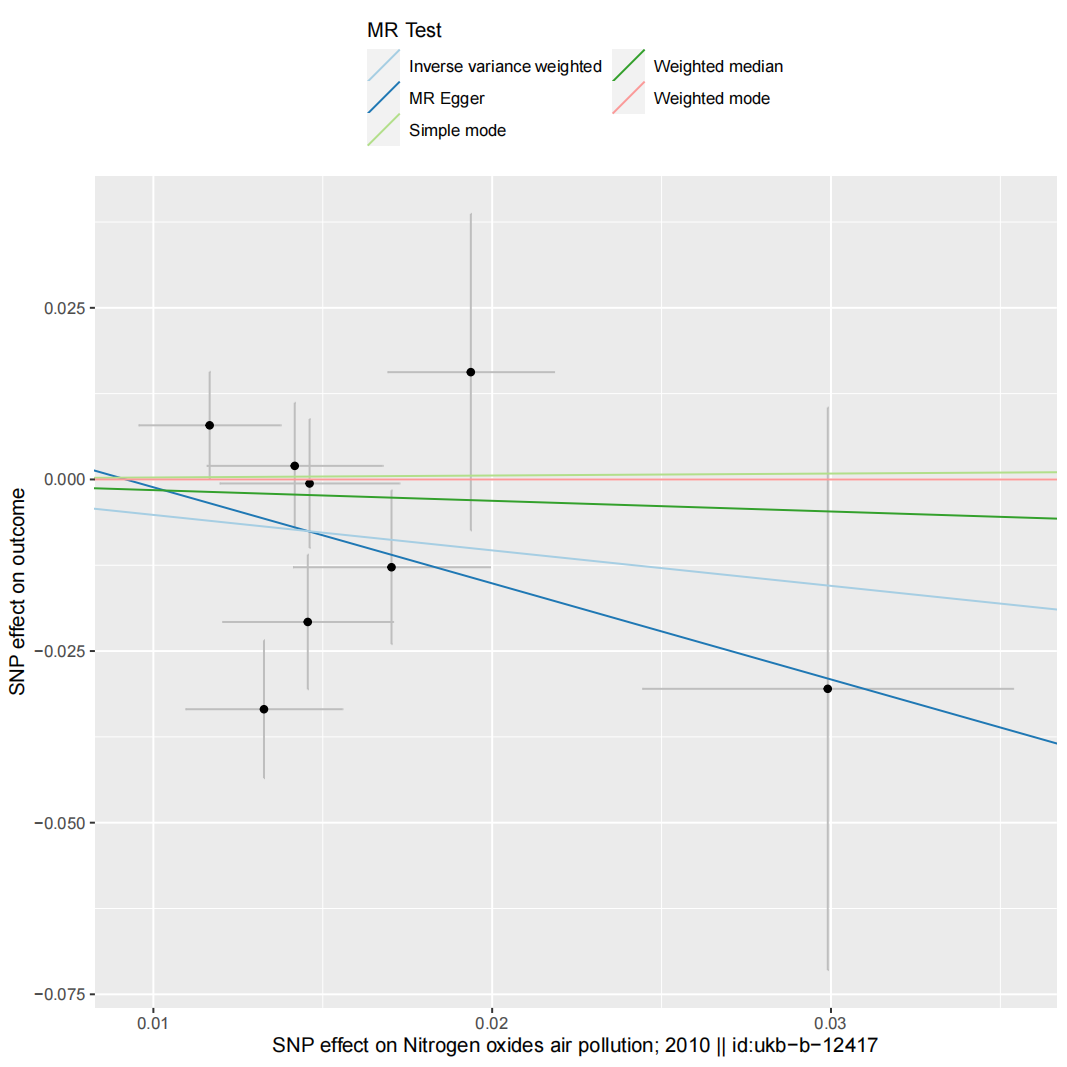

Supplement: Supplementary file 4 [file Image_3.TIF]

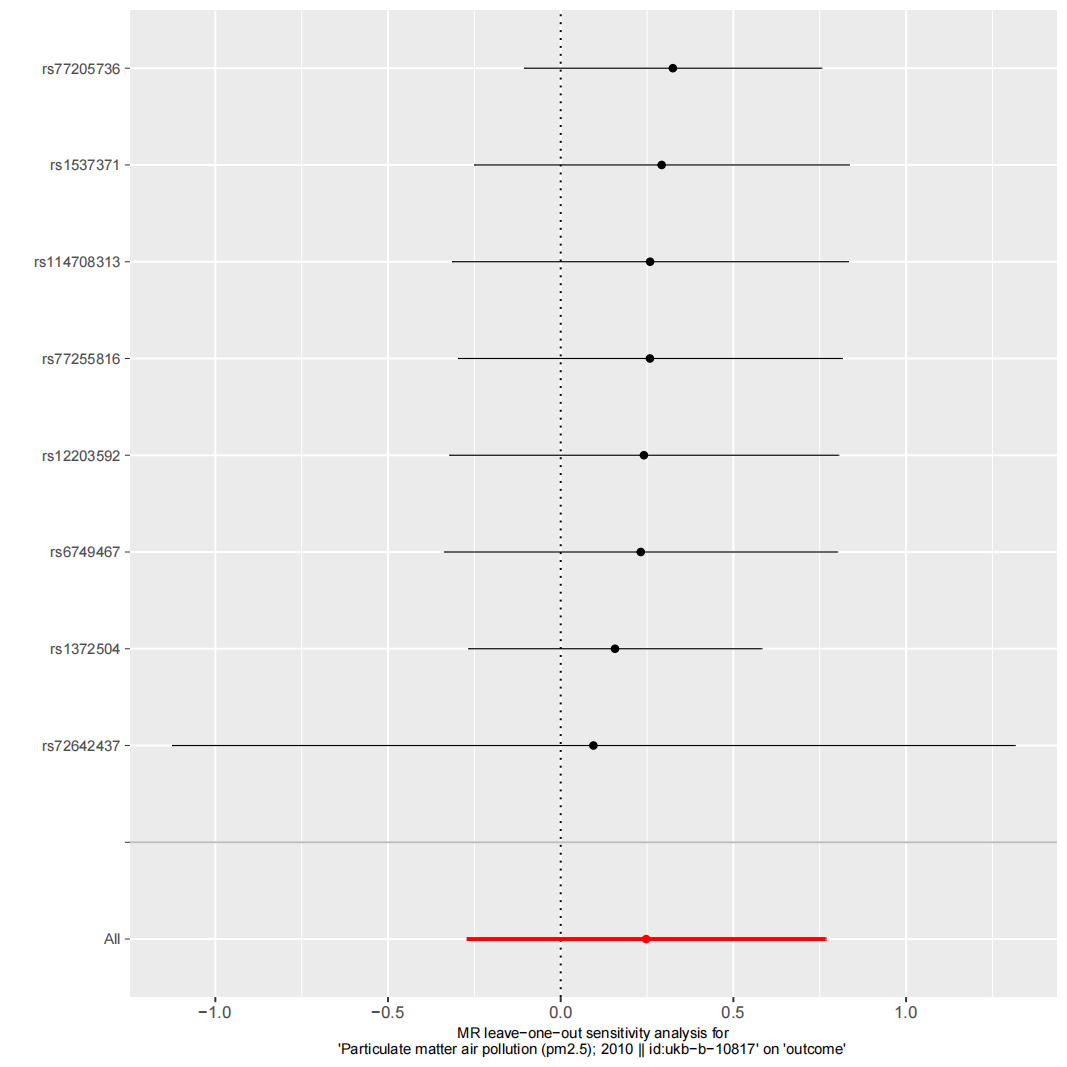

Supplement: Supplementary file 5 [file Image_4.TIF]

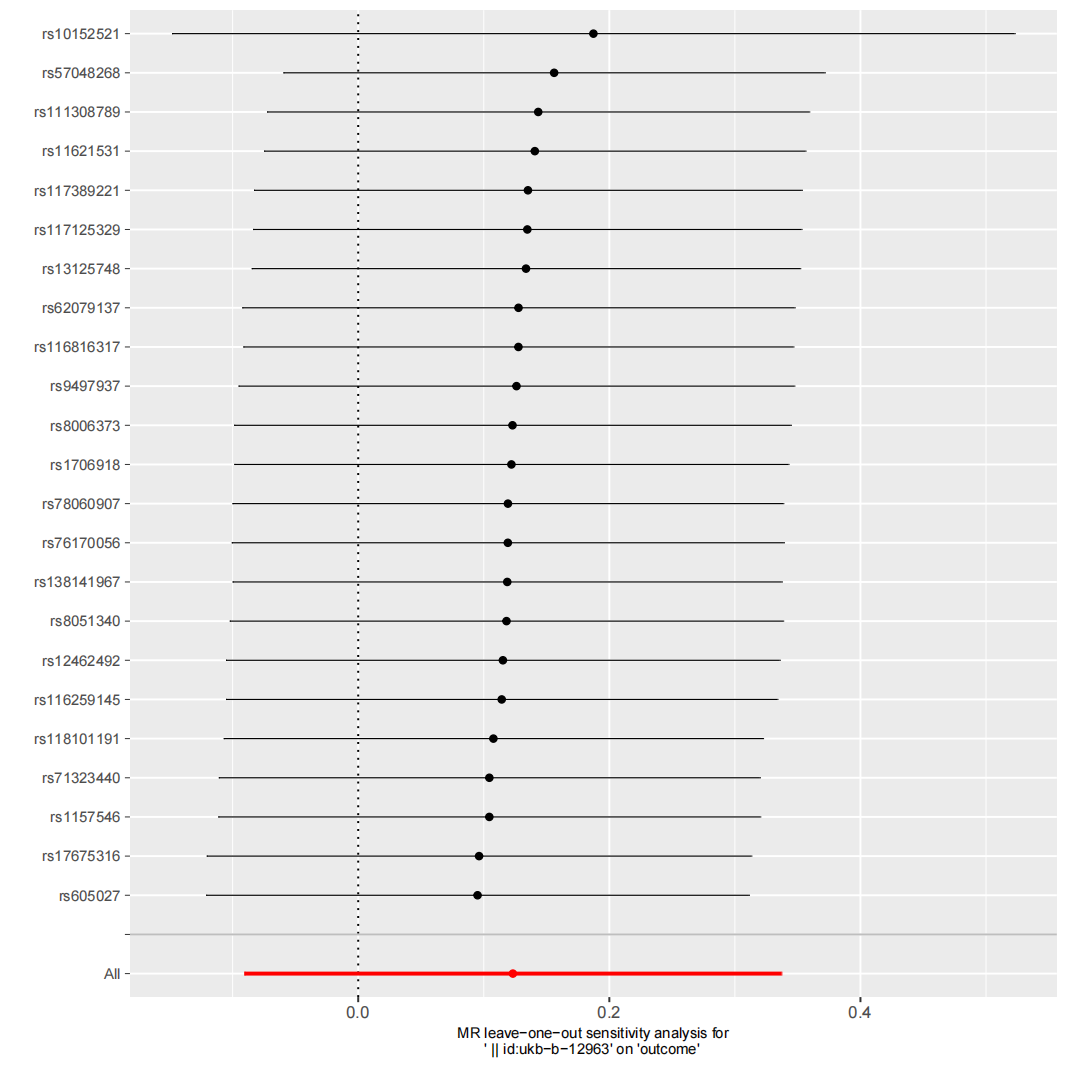

Supplement: Supplementary file 6 [file Image_5.TIF]

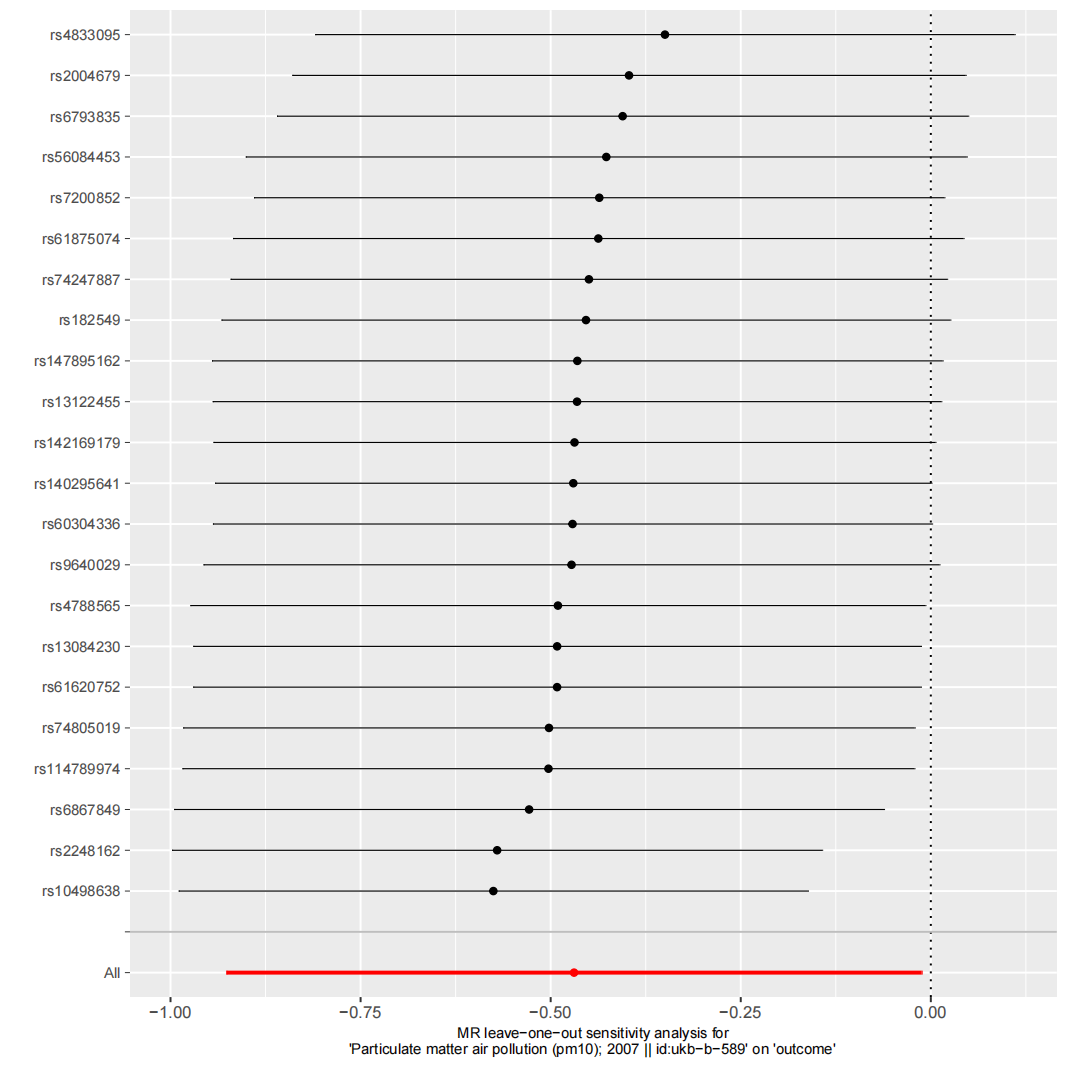

Supplement: Supplementary file 7 [file Image_6.TIF]

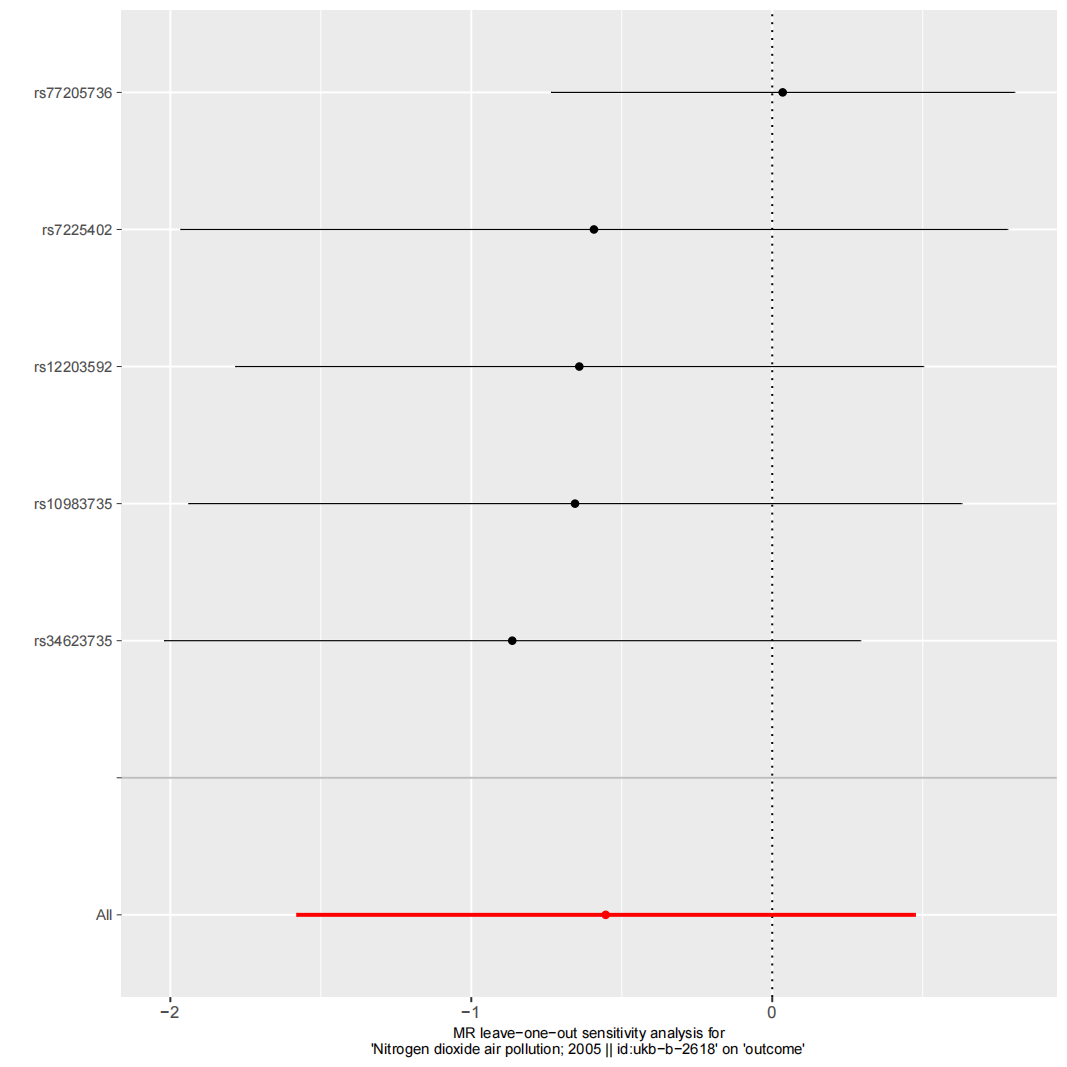

Supplement: Supplementary file 8 [file Image_7.TIF]

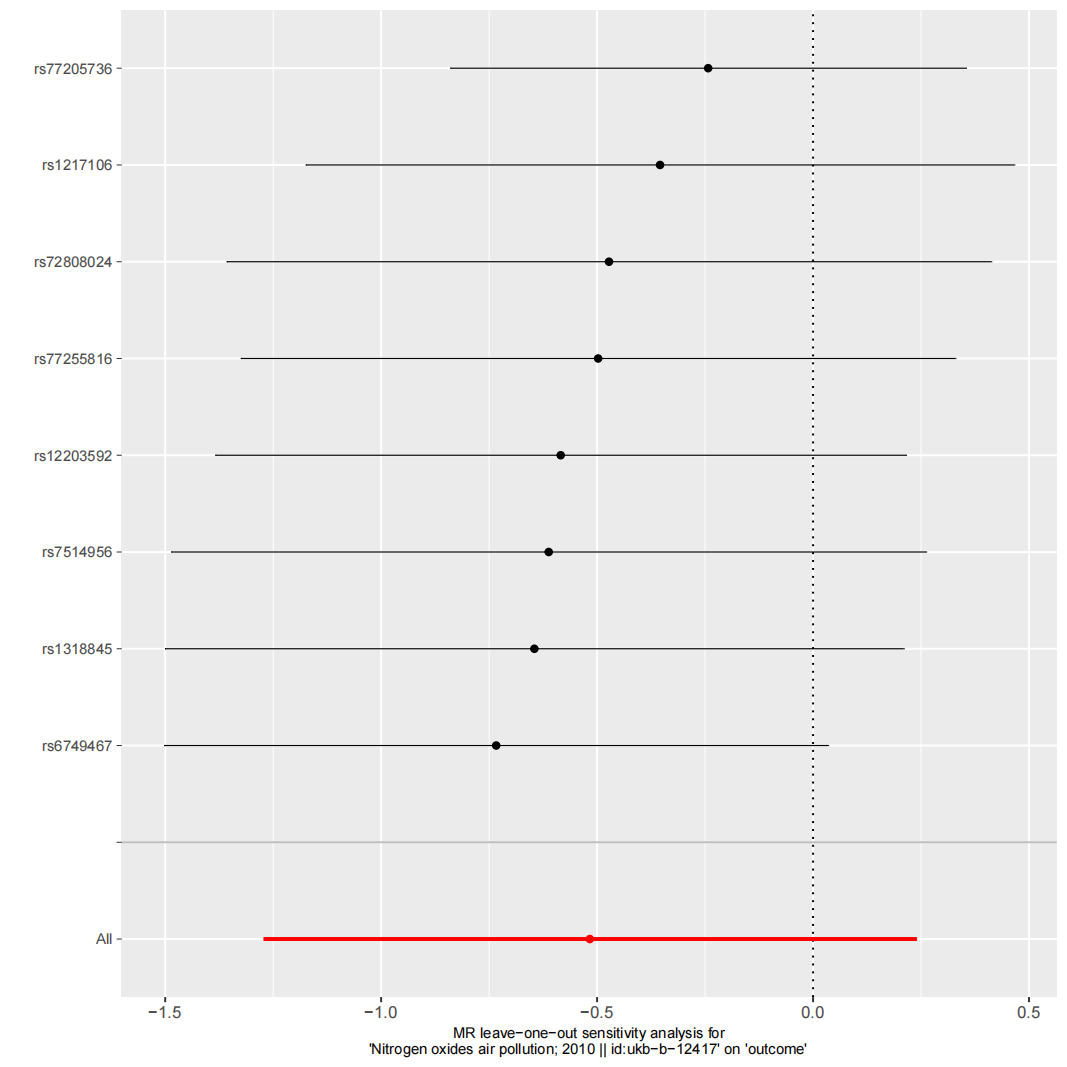

Supplement: Supplementary file 9 [file Image_8.TIF]
